# CLIMATE BRAIN - Questionnaires, Tasks and the Neuroimaging Dataset

**DOI:** 10.1038/s41597-025-05038-0

**Published:** 2025-05-01

**Authors:** Dominika Zaremba, Bartosz Kossowski, Marek Wypych, Katarzyna Jednoróg, Jarosław M. Michałowski, Christian A. Klöckner, Małgorzata Wierzba, Artur Marchewka

**Affiliations:** 1 Laboratory of Brain Imaging, Nencki Institute of Experimental Biology, Polish Academy of Sciences, Warsaw, Poland; 2 Laboratory of Language Neurobiology, Nencki Institute of Experimental Biology, Polish Academy of Sciences, Warsaw, Poland; 3 Poznan Laboratory of Affective Neuroscience, SWPS University, Institute of Psychology, Warsaw, Poland; 4 Department of Psychology, Norwegian University of Science and Technology, NTNU, Trondheim, Norway

**Keywords:** Climate change, Psychology and behaviour, Climate-change mitigation, Human behaviour, Interdisciplinary studies

## Abstract

Climate change threatens human populations and ecosystems worldwide. Neuroscience research on this topic is emerging, but validated questionnaires, stimuli, and fMRI tasks remain scarce. Here, we present the CLIMATE BRAIN dataset, a multimodal collection of questionnaire, behavioral, and neuroimaging data from 160 young, healthy Polish individuals. Designed to advance research on climate emotions and pro-environmental behavior, the dataset includes individuals with moderate climate change concern. Participants read anger and hope-evoking stories about climate change and made pro-environmental decisions. The dataset includes data from (1) various questionnaire measures, including the Inventory of Climate Emotions (ICE); (2) a neuroimaging task for measuring emotional reactions to standardized Emotional Climate Change Stories (ECCS); and (3) a neuroimaging task based on Carbon Emission Task (CET) to measure climate action-taking. For technical validation, we provide image quality metrics and show the evidence for the effectiveness of the tasks consistent with prior studies. To our knowledge, the proposed multimodal dataset is currently the only publicly available resource specifically designed to investigate human brain responses to climate change.

## Background & Summary

Climate change poses a significant and immediate threat to both human populations and ecosystems worldwide^1^. Despite clear expert recommendations, the pace of necessary systemic changes remains inadequate. Understanding human responses to climate change is crucial for fostering a bottom-up, individual behaviour change, which is just as necessary as legal and technological solutions^2^. Although many disciplines have contributed to this understanding, neuroscience can offer insights into the brain mechanisms that drive emotional reactions to climate change and pro-environmental decision-making^3^.

One promising line of research involves the role of emotions related to climate change (henceforth: climate emotions) in the formation of attitudes and behaviours. Strong emotions, especially climate anger and climate hope have been the focus of researchers investigating drivers of environmental activism^4–6^, while climate anxiety has been identified as an important factor for youth mental health^7,8^. A better understanding of climate emotions can inform more effective strategies to motivate individuals and communities to engage in sustainable practices^9^. Affective neuroscience can provide another layer of explanation and bring new insights into the research and practice.

Neuroscience can also contribute to the understanding of the brain processes that stay behind individual climate-friendly or unfriendly choices. Many studies on pro-environmental behaviour and climate-related decision-making have traditionally relied on measures that, while cost-effective and convenient, often lack validity. Conclusions about the effectiveness of interventions had to be formulated based on self-report measures of intentions, and attitudes^10^. Unfortunately, declarations and decisions made in hypothetical scenarios often poorly reflect real-life behaviours^11,12^. Thus, significant challenges persist in developing behavioural measures with higher ecological validity.

Given that the efforts of environmental neuroscientists are pioneering, advancing this field requires adherence to open science practices, such as sharing validated research tools, stimuli and reliable data. While several studies have explored how specific brain circuits relate to pro-environmental behaviours and judgments^3,13^, few publicly available datasets exist^14^. Therefore, we present the CLIMATE BRAIN dataset, a unique resource derived from 160 healthy participants designed to advance future research on the neural basis of climate emotions and decision-making.

CLIMATE BRAIN contains data from multiple sources, including the Inventory of Climate Emotions (ICE^15^) and other questionnaire measures, a neuroimaging task Reading and Rating Emotional Stories (RRES) designed to assess emotional responses to standardized Emotional Climate Change Stories (ECCS^16^), and the Carbon Emission Task (CET^17^), adapted to functional magnetic resonance imaging (fMRI) settings.

The CLIMATE BRAIN dataset can be used to explore how brain responses to emotional climate stories relate to subjective experience (RRES task). For instance, researchers can investigate differences in brain responses to stories evoking different emotions. Because subjective ratings of emotional stimuli were also recorded, it is possible to analyze whether the brain activity can be predicted by declarative ratings of valence and arousal. Secondly, CLIMATE BRAIN can offer insights into how individuals balance competing values in the context of climate action (CET task). The dataset can be used to investigate how individuals weigh immediate personal rewards and future environmental costs. Because these parameters were manipulated independently, it is possible to model the subjective value of the choice options and test whether it can predict climate friendly choices^18^.

The main limitation that should be considered when using the CLIMATE BRAIN dataset is the demographic composition of the studied sample. In particular, we recruited individuals who express moderate climate change concern. Furthermore, the sample was quite homogenous in terms of age (young adults), place of residence (urban) and education (at least secondary). While this limits generalizability, the data still shows meaningful individual variability in behaviour and brain activity.

To the best of our knowledge, the proposed dataset is currently the only openly available source of data and tasks specifically designed to explore the relationship between emotion, pro-environmental decision-making, and other psychological, theoretically related individual characteristics, such as the level of climate change concern, sense of efficacy, socioeconomic status or political affiliation, on both behavioural and brain level.

## Method

### Subject characteristics

The study was conducted in accordance with the Declaration of Helsinki and was approved by the Ethical Review Board of the SWPS University in Poland (approval no. 2023-167). All participants provided written informed consent and signed an extended document detailing study information, including data privacy and the processes of pseudo-anonymization and anonymization for analyses and publications related to the research project. The consent form was inspired by the Open Brain Consent template^19^. The public dataset is now fully anonymized, ensuring the privacy and confidentiality of all participants.

The sample comprised 160 individuals aged 19 to 26 years (M = 22, SD = 1.76) evenly split between men and women (80 each). Participants were recruited through a large, online panel managed by an external company. The recruitment was based on specific inclusion criteria: at least a high school education, no history of neurological or psychiatric disorders, right-handedness, no contraindications to MRI scanning, residence in Poland for most of the past five years and Polish as first language. Additionally, the participants were required to exhibit a moderate level of concern about climate change, as indicated by their response on a scale from 0 to 4, where moderate concern was defined as a score of 1, 2, or 3. This approach helped exclude those with very low concern, who tend to resist pro-environmental behaviour, and those with high concern, who engage in it regardless—both of which could reduce sensitivity to intervention effects^20^. Moderately concerned individuals are more likely to adjust their behaviour in response to interventions^21^. Although this approach narrows the range of concern, our sample remains representative of 80% of Poland’s population^22^ and exhibits meaningful variability in decision-making. To prevent selection bias and avoid recruiting individuals with extreme concern, we embedded climate-related questions within a broader survey on unrelated topics. Participants received a fixed remuneration of 200 Polish zloty (PLN), approximately 46 EUR. However, in order to introduce experimental manipulation, participants were initially informed that they would receive a guaranteed 120 PLN, but their total remuneration could increase up to 240 PLN depending on their decisions in one of the tasks (see: Carbon Emission Task).

The gender, year of birth, place of residence, and education level of the participants were recorded. We also measured numerous variables that are theoretically related to climate emotion and decision-making measured in the current study, i.e. political affiliation, perceived socioeconomic status, driver’s license status, car usage frequency as a passenger or a driver, concern about climate change, climate action efficacy beliefs, perceived eco-friendliness of one’s lifestyle, perceived importance of individual or collective climate action, psychological distance to climate change, nature relatedness and baseline level of 8 climate emotions. Participants completed a health screening survey to ensure their safe participation in the experiment. The data from the health screening survey are not publicly available.

### Image acquisition

Neuroimaging data were acquired on a 3-Tesla Siemens Prisma scanner with a 32-receive channel head coil. An anatomical T1-weighted scan was acquired at the beginning of the scanning session using a magnetization-prepared rapid gradient-echo sequence (MPRAGE) with a voxel size of 1 × 1 × 1 mm isotropic (field of view = 256 × 176 × 256 mm [A-P; R-L; F-H]) in sagittal orientation. In the case of three participants, due to insufficient quality a T1-weighted scan had to be acquired for the second time at the end of the scanning session. Functional data were acquired using echo-planar imaging pulse sequence (multi-band acceleration factor 2, in-plane acceleration factor 2, repetition time [TR] = 2000 ms, echo time [TE] = 30 ms, flip angle [FA] = 70°). We acquired 66 slices in transverse plane orientation with an isotropic voxel size of 2.5 × 2.5 × 2.5 mm. For estimating magnetic field inhomogeneities, we additionally acquired two spin-echo EPIs with an inverted phase-encoding direction. The acquired DICOM files were converted to NIfTI and organized according to Brain Imaging Data Structure (BIDS) Specification version 1.8.035 using dcm2bids version 3.2.0^23^.

### Image processing

The CLIMATE BRAIN dataset contains both raw and preprocessed data. Anatomical images have been anonymized by removing facial features using mri_deface, 1.22^24^. The released preprocessed data comes from the preprocessing performed using fMRIPrep, 24.1.0^25,26^; RRID:SCR_016216, which is based on Nipype 1.8.6^27,28^, RRID:SCR_002502.

A B0-nonuniformity map was estimated based on two echo-planar imaging (EPI) references with topup^29^ in FSL^30^, version 6.0.7.7, RRID:SCR_002823. T1w images were corrected for intensity non-uniformity (INU) with N4BiasFieldCorrection^31^, distributed with ANTs^32^, version 2.5.1, RRID:SCR_004757 and used as T1w-reference throughout the workflow. The T1w-reference was then skull-stripped with a Nipype implementation of the antsBrainExtraction.sh workflow (from ANTs), using OASIS30ANTs as target template. Brain tissue segmentation of cerebrospinal fluid (CSF), white-matter (WM) and grey-matter (GM) was performed on the brain-extracted T1w using fast FSL. Volume-based spatial normalization to one standard space (MNI152NLin2009cAsym) was performed through nonlinear registration with antsRegistration (ANTs), using brain-extracted versions of both T1w reference and the T1w template. The following template was selected for spatial normalization and accessed with TemplateFlow^33^, 23.1.0,: ICBM 152 Nonlinear Asymmetrical template version 2009c^34^ [RRID:SCR_008796; TemplateFlow ID: MNI152NLin2009cAsym].

For each of the 4 BOLD runs found per subject (across all tasks and sessions), the following preprocessing was performed. First, a reference volume was generated, using a custom methodology of fMRIPrep, for use in head motion correction. Head-motion parameters with respect to the BOLD reference (transformation matrices, and six corresponding rotation and translation parameters) are estimated before any spatiotemporal filtering using mcflirt (FSL^35^). The estimated fieldmap was then aligned with rigid registration to the target EPI (echo-planar imaging) reference run. The field coefficients were mapped onto the reference EPI using the transform. The BOLD reference was then co-registered to the T1w reference using mri_coreg (FreeSurfer) followed by flirt (FSL^36^) with the boundary-based registration^37^ cost-function. Co-registration was configured with six degrees of freedom. Several confounding time series were calculated based on the preprocessed BOLD: framewise displacement (FD), DVARS and three region-wise global signals. The three global signals are extracted within the CSF, the WM, and the whole-brain masks. Additionally, a set of physiological regressors was extracted to allow for component-based noise correction (CompCor^38^). Frames that exceeded a threshold of 0.5 mm FD or 1.5 standardized DVARS were annotated as motion outliers. The preprocessed functional images were then smoothed with a 6-mm FWHM Gaussian kernel within SPM12 (Wellcome TrustCentre for Neuroimaging, University College, London, UK, http://www.fil.ion.ucl.ac.uk/spm/software/spm12) running on MATLAB2023a (MathWorks, http://www.mathworks.com).

### Dataset validation

Behavioural data analysis and visualisation were performed using R^39^ (Version 4.1.2) in RStudio^40^ (Version 2022.12.0, RRID: SCR_000432). Neuroimaging data quality measures were extracted with MRIQC software^41^. Neuroimaging data analysis was performed using SPM12 (Wellcome TrustCentre for Neuroimaging, University College, London, UK, http://www.fil.ion.ucl.ac.uk/spm/software/spm12) running on MATLAB2023a (MathWorks, http://www.mathworks.com). Additionally, the BIDS-Matlab^42^ (RRID:SCR_022292) software was used to preprocess the confounds timeseries generated by fMRIprep. Finally, the neuroimaging results were visualized using the Nilearn^43^ (RRID:SCR_001362) toolbox for Python.

### Experimental design

**Fig. 1:**
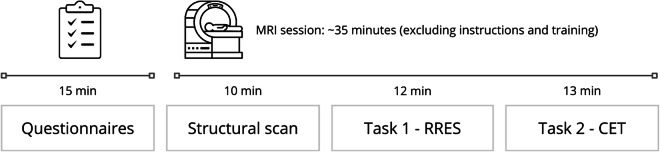
The overview of the experimental protocol of the CLIMATE BRAIN dataset.

Before the MRI session, participants completed a demographic survey and a set of questionnaires, as well as an MRI safety survey. The MRI session began with the localizer, shim and T1-weighted scan sequences. After a brief instruction, they completed a training session followed by three target runs of the **Reading and Rating Emotional Stories** (RRES) task. Then participants read detailed instructions for the **Carbon Emission Task** (CET) at their own pace. After answering comprehension questions and completing a training session, they completed the target CET run. In the end, a fieldmap scan was acquired. All instructions, training sessions and both experimental tasks were administered using Presentation software (Version 23.0, Neurobehavioural Systems, Inc., Berkeley, CA). The whole experimental session lasted approximately 1,5 hours, including an 1-hour-long MRI scan (including instruction and training time). Figure 1 presents the overview of the experimental protocol.

#### Questionnaires

Participants completed the Personal and Collective Action Efficacy^44^, Psychological Distance to Climate Change^45^, Nature Relatedness^46^, and Inventory of Climate Emotions^15^ questionnaires, and answered additional custom questions (see full list in the Supplementary Materials) outside of the scanner. A list of measured constructs along with example items can be found in Table 1.

**Table 1:** List of the questionnaire measures included in the CLIMATE BRAIN dataset.

Questionnaire	Construct	Example items
Demographic (1 item per construct, except for Political affiliation investigated with 3 conditional questions)	Sex	*Indicate your sex. (multiple options)*
	Age	*Indicate your age. (input field)*
	Residence	*Which of the following best describes the area you live in? (multiple options)*
	Education	*What is your educational attainment? (multiple options)*
	Climate change concern	*How concerned are you about climate change?* *Scale < 0–4, Not at all concerned — Extremely concerned>*
	Relative importance of collective vs. individual climate actions	*What actions do you consider most important in addressing climate change?* *Scale < 1–5, Collective actions — Individual actions>*
	Perceived climate friendliness	*Compared to other people, do you consider your lifestyle…?* *Scale < 1–5, Definitely climate-unfriendly — Definitely climate-friendly>*
	Driving licence ownership	*Do you have a driving licence? (Yes/No)*
	Car usage	*How often do you use a car (e.g. as a driver or a passenger)?* *Scale < 0–3, Once a year or several times a year — Everyday>*
	Political affiliation	*Do you have any political views? (Yes/No)* *If yes:* *In politics, the terms /left/ and /right/ are used. Can you describe your political views using these terms? (Yes/No)* *If yes:* *Please indicate your political views*. *Scale < 0–10, Left — Right>*
	Socioeconomic status	*Which of the descriptions below comes closest to how you feel about your household income nowadays?* *Scale < 0–3, Living comfortably on present income — Finding it very difficult on present income>*
PCAE^44^ (4 items, 5-point scale from Strongly disagree to Strongly agree)	Individual climate action efficacy	*I believe my actions can have a beneficial influence on climate change*.
	Collective climate action efficacy	*If we act collectively, we will be able to minimize the consequences of climate change*.
PD^45^ (2 items, 7-point scale from Strongly disagree to Strongly agree)	Psychological distance to climate change - temporal	*It will be a long time before the consequences of climate change are felt*.
	Psychological distance to climate change - geographical	*My local area will be influenced by climate change*.
NR^46^ (6 items, 5-point scale from Disagree strongly to Agree strongly)	Nature relatedness	*My relationship to nature is an important part of who I am*.
ICE^15^ (32 items, 5-point scale from Strongly disagree to Strongly agree)	Anger	*I feel angry that the political and economic system that we live in harms the climate*.
	Contempt	*I am annoyed by the constant publicity around climate change*.
	Enthusiasm	*The increasing public engagement with climate change gives me hope*.
	Powerlessness	*I am overwhelmed by how many aspects of life would need to be changed to limit climate change*.
	Guilt	*It upsets me that I have a big negative impact on the climate*.
	Isolation	*I feel like one of the few people who actually understand what climate change entails*.
	Anxiety	*Thinking about climate change makes me fear for the future of our children*.
	Sorrow	*I am sad that so many living creatures suffer because of climate change*.

#### Reading and Rating Emotional Stories (RRES)

The task was designed to measure brain activity related to experiencing climate emotions. We recorded brain activity while participants read short stories and rated their emotional response to them on the scales of valence and arousal. The stories were selected from the Emotional Climate Change Stories (ECCS) dataset^16^, which was previously found to be effective in evoking climate emotions. ECCS stories were created on the basis of qualitative research on climate emotions and the context in which they most commonly emerge^47^, and rated on the scales of valence, arousal, anger, anxiety, compassion, guilt and hope in the course of three independent studies^16^. To select stories we employed a classification method developed by our team^16,48^. Specifically, we used a simple genetic algorithm to identify stories that best represent each emotion category: anger (ANG), hope (HOP) and neutral (NEU). The control (CON) stories were drawn from the same pool as NEU stories. No statistically significant differences in story length across story types were observed (ANG: *M* = 274, *SD* = 53.2; HOP: *M* = 309, *SD* = 40.6; NEU: *M* = 305, *SD* = 53.2; CON: *M* = 278, *SD* = 35.1; *F*(3, 44) = 1.80, *p* = 0.16) Example stories can be found in Table 2.

**Table 2:** Example stories from the Emotional Climate Change Stories dataset used in the RRES task.

Example Stories	
Anger	A corporation commissioned a report on its environmental impact. As the report turned out to embarrass the corporation, the management decided to conceal it. Employees were forbidden to disclose any information on this subject.
Hope	Amara is an engineer and she specialises in fire safety. She has invented a special coating for buildings to protect them from fires, which are increasingly common. What is more, the coating is made of industrial waste, which would normally end up in a landfill.
Neutral	John entered the conference room and sat down on a chair. Only then did he look around and think, “If the front row seats have not all been taken yet, I’ll switch.” He got up and moved closer to the screen at the front of the room.
Control	George decided to take a break from work and have some lunch. He usually eats a bun, but this time he only had some bread at home. He cut two slices, took the rest of the ingredients out of the refrigerator, and made sandwiches. He took the plate with the sandwiches to his desk and ate them there as he browsed a news website.

**Fig. 2:**
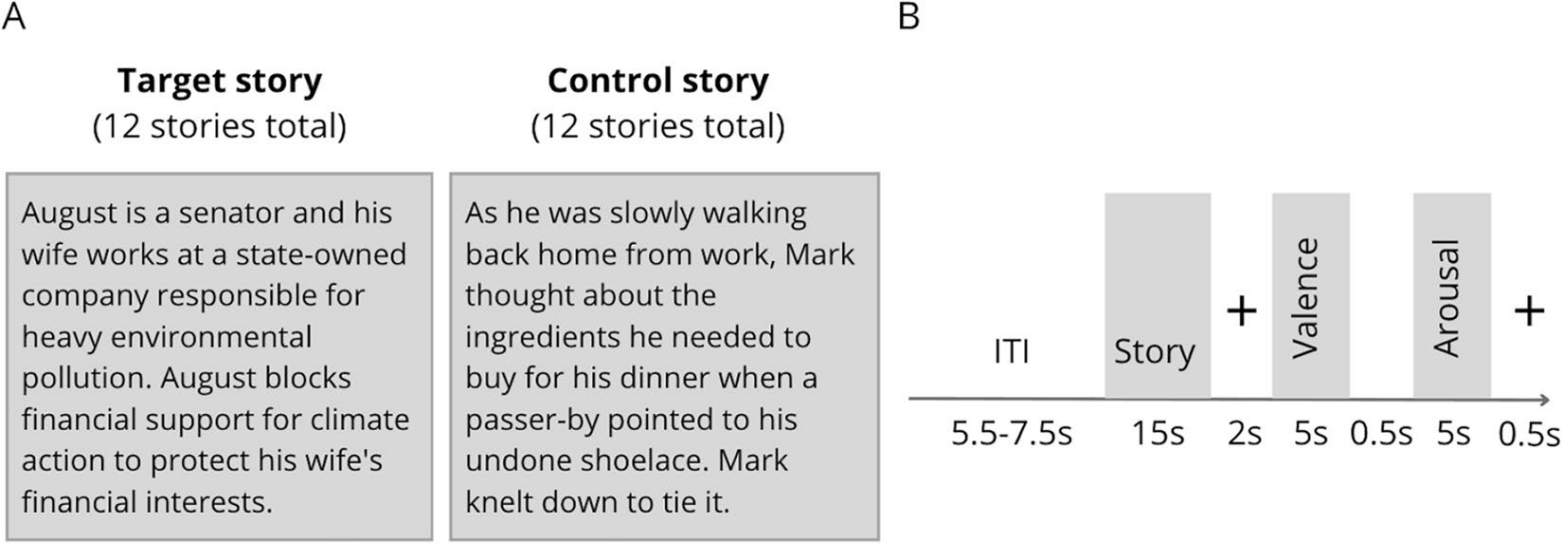
Reading and Rating Emotional Stories task overview. (**A**) Participants read short stories about people and their actions. In ANG and HOP groups, target stories were related to climate change and aimed to evoke anger and hope, respectively. In the NEU group, target stories described everyday situations without emotional content. Here, we present an example of an anger-eliciting story and a control story. (**B**) Stimuli were presented with jittered inter-trial intervals. Each trial consisted of two phases: the story-reading phase and the rating phase.

Participants were pseudo-randomly assigned to three groups, each exposed to stories inducing either anger (ANG, n = 54), hope (HOP, n = 54), or a neutral state (NEU, n = 52). Each participant read 12 target stories and 12 control stories evoking a neutral state. In ANG and HOP groups, target stories were related to climate change and aimed to evoke anger and hope, respectively. In the NEU group, target stories described everyday situations without emotional content. Control stories used across all groups were identical, depicting everyday situations without emotional content. Stories were presented in three runs, with each comprising 8 stories (4 target, TAR and 4 control, CON) in a fixed, pseudo-random order. Each trial began with a 15-second presentation of a story, followed by a 2-second display of a fixation cross. After each story, participants were instructed to rate their emotions on two scales in the following order: valence ranging from negative to positive emotions on an 11-point scale, and arousal, ranging from low to high arousal on another 11-point scale. They used a two-button response pad to move the cursor on the valence and arousal scales. They had 5 seconds to provide each rating. Trials were separated by a jittered intertrial interval (ITI) lasting between 5.5 and 7.5 seconds. An overview of the RRES is presented in Fig. 2.

Although the RRES task has great usability in the fMRI environment, it also has some limitations. First, while using written stories allowed us to overcome the challenges posed by the loud scanner environment and how it impacts stimulus delivery, this format may not fully capture the emotional impact that more immersive formats (e.g., audio or video) of stimuli might evoke.

#### Carbon Emission Task (CET)

When selecting the task to measure pro-environmental behaviour, we considered several well-documented experimental paradigms, including those measuring donations, product choices, recycling, resource consumption, and social dilemma games^49^. We excluded tasks where performance affected experiment duration (such as Work for the Environment Protection Task^50^ and Pro-Environmental Behaviour Task^10^) to ensure a consistent number of volumes acquired during the fMRI session^10,50^. Additionally, paradigms like FISH 3^51^ or the Greater Good Game^52^, which focus on the influence of social feedback (e.g., others’ choices), were not used, as they did not align with our research goals. The Carbon Emission Task, developed by Berger and Wyss^17^, involves participants making a series of decisions between receiving a monetary bonus (a self-interest choice) and retiring carbon emission certificates (a climate-friendly choice). Choosing the climate-friendly option reduces the number of certificates available for purchase in Emission Trading Scheme. Emission Trading is an effective strategy for limiting the amount of carbon emissions^53^, which makes the CET task an ecologically valid way to measure pro-environmental behaviour related to climate change.

We adapted the original CET task for fMRI settings by: (1) reducing the visual complexity of the task; (2) choosing stimuli presentation time and inter-trial intervals to maximize the signal-to-noise ratio; (3) increasing the number of trials; (4) adjusting the possible monetary bonus and carbon emission levels; (5) adding dummy trials to control for motor activity (“Choose the option on the left/right”). Despite these design changes, the CET task still has some limitations. First, it does not include a control condition for decision-making that is unrelated to climate issues, which makes the results harder to interpret. It is impossible to determine whether the results are specific to the context of climate change or if they reflect decision-making in general. Second, while the task helps estimate how much participants value money compared to reducing emissions, it does not tell us whether their choices are driven more by personal benefit or by caring about the environment.

**Fig. 3:**
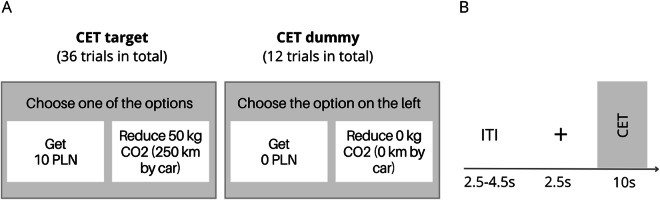
Carbon Emission Task overview. (**A**) Participants made a series of decisions between a monetary bonus and a reduction of CO_2_ emissions. (**B**) Stimuli were presented with jittered inter-trial intervals. Each trial began with a fixation cross, followed by a binary choice between a monetary bonus or carbon emission reduction.

Data collection was carried out in a single run consisting of 48 trials (36 target trials and 12 dummy trials). Each trial began with a 2.5-second display of a fixation cross, followed by a 10-second presentation of the stimulus. Trials were separated by a jittered intertrial interval (ITI) lasting between 2.5 and 4.5 seconds. In the target trials, participants were instructed to select either a financially rewarding or climate-friendly option using a two-button response pad. Available options were different in each trial: monetary bonus of 0, 10, 50, 80, 100, or 120 Polish zloty (PLN; 1 PLN ~ 0.25 EUR) versus a reduction of CO_2_ emissions by 0, 2, 10, 25, 40, or 50 kg CO_2_. In dummy trials, the value of options was 0 PLN and 0 kg of CO_2_. Participants’ task was to follow the instruction on the top of the screen and select the option on the left or the right (Fig. 3). Among the 36 target trials, 10 were “rational trials” in which one of the options presented was 0. These trials involved choices between (a) receiving some monetary bonus (e.g., 100 PLN) with no reduction in CO_2_ emissions (0 kg), or (b) no monetary bonus (0 PLN) but with some reduction in CO_2_ emissions (e.g., 20 kg). These rational trials were included to ensure that participants made decisions in a consistent, rational manner, as the option with the higher payoff was objectively better. To ensure that participants treated each decision independently, they were told that one of their choices would be randomly selected to determine whether they receive a bonus or retire carbon emission certificates. In reality, all participants received a fixed bonus of 80 PLN on top of their base remuneration of 120 PLN. Additionally, we eliminated 25 kg of CO_2_ on behalf of each participant.

## Data Records

This data descriptor outlines the behavioural and neuroimaging dataset available on OpenNeuro^54^ under the accession number ds005460 (10.18112/openneuro.ds005460.v2.0.0). The dataset is organised following the Brain Imaging Data Structure (BIDS) Specification version 1.8.035. Additionally, the behavioural data is also available on the Open Science Framework (10.17605/OSF.IO/ZEQNX)^55^.

The participants.tsv file contains the demographic and questionnaire data of each participant. The accompanying participants.json sidecar file provides the metadata necessary to understand its content.

Additionally, the repository includes subject-wise *_events.tsv files, containing behavioural data for each task, along with corresponding *_events.json sidecar files that provide metadata necessary to understand their content.

The neuroimaging data includes both raw and preprocessed images. Anatomical images have been defaced to protect the anonymity of study participants.

The derivatives directory contains MRIQC and fMRIprep output. In the case of MRIQC output, we share the summary file group_bold.tsv, which contains image quality metrics for all participants. In the case of fMRIprep output, we share subject-wise data organized in sub-* folders with preprocessed functional data, confound time series (allowing for motion and physiological noise correction), individual brain masks for each task and session, as well as accompanying .json sidecar files. These derivative files are named in the following convention:

- All files related to the RRES task contain the task-stories label in the filename.
- Additionally, files related to the respective sessions of the RRES task contain a run label: run-01, run-02, run-03 in the filename.
- All files related to the CET task contain the task-cet label in the filename.

Supporting materials such as checklists used during experimental procedures, full demographic survey, questionnaires and task instructions are available in Supplementary Materials.

## Technical Validation

### Data collection and experiment design

The emotional stimuli come from a previously validated Emotional Climate Change Stories dataset^16^. The Carbon Emission Task’s validity and reliability have also been already established^17^. To ensure that the participants understood task instructions, we included a training session and comprehension questions in both RRES and CET tasks. To allow within- and between-group analyses, we included a control neutral group (NEU, n = 52) as well as control stimuli in each task. Personnel responsible for the data acquisition received detailed training and were required to check instruction comprehension during the data collection process (see Supplementary Materials).

### Exclusion criteria

We used MRIQC software to assess image quality, focusing on metrics such as temporal signal-to-noise ratio (tSNR) and framewise displacement (FD). We excluded participants with runs with a temporal signal-to-noise ratio (tSNR) below 40 or where more than 15% of volumes had framewise displacement (FD) exceeding 0.5 mm, as these thresholds are widely used to identify data with poor signal quality or excessive motion^56,57^. Additionally, we identified a run in which technical issues compromised data acquisition and excluded one participant on this basis. This updated criterion results in the exclusion of 6 participants in the RRES task (sub-2112b, sub-2911e, sub-0712b, sub-0712c, sub-1512d, sub-1512a) and 6 participants in the CET task (sub-2112b, sub-2911e, sub-0712b, sub-0712c, sub-1312d, sub-3011a) from the validation analyses presented below. Nevertheless, we share data from these participants if other researchers would like to use different exclusion criteria.

The original output table from MRIQC is available in the OpenNeuro repository. The summary table with descriptive statistics about image quality is available in the Supplementary Materials.

### Behavioural and neuroimaging data

**Fig. 4:**
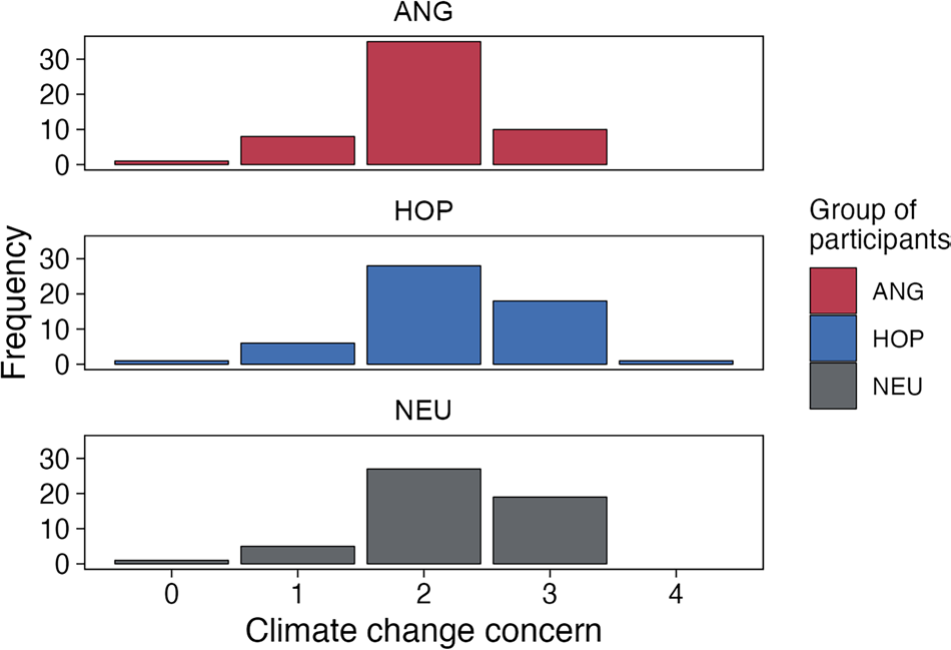
Climate change concern in each group. Participants answered the question: “How concerned are you about climate change?” on a scale from “Not at all concerned” (0) to “Extremely concerned” (4).

Participants from all groups exhibited a similar climate change concern before the experimental manipulation (Fig. 4). We found no evidence for the group differences in mean climate change concern (F(2,157) = 1.88, p = 0.156).

**Fig. 5:**
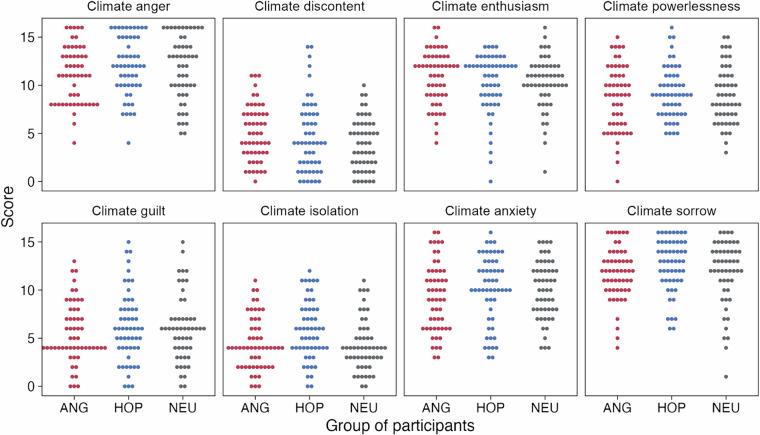
The profile of climate emotions assessed with the Inventory of Climate Emotions (ICE) in each group. Dots represent individual summary scores on each of the ICE subscales. Each subscale consists of 4 items, rated on a scale from 0–4.

In all groups participants exhibited a comparable profile of climate emotions (Fig. 5), as indicated by separate one-way ANOVAs for each emotion subscale (all Fs < 4.66, all adjusted ps > 0.19, after Bonferroni correction).

#### RRES validation

**Fig. 6:**
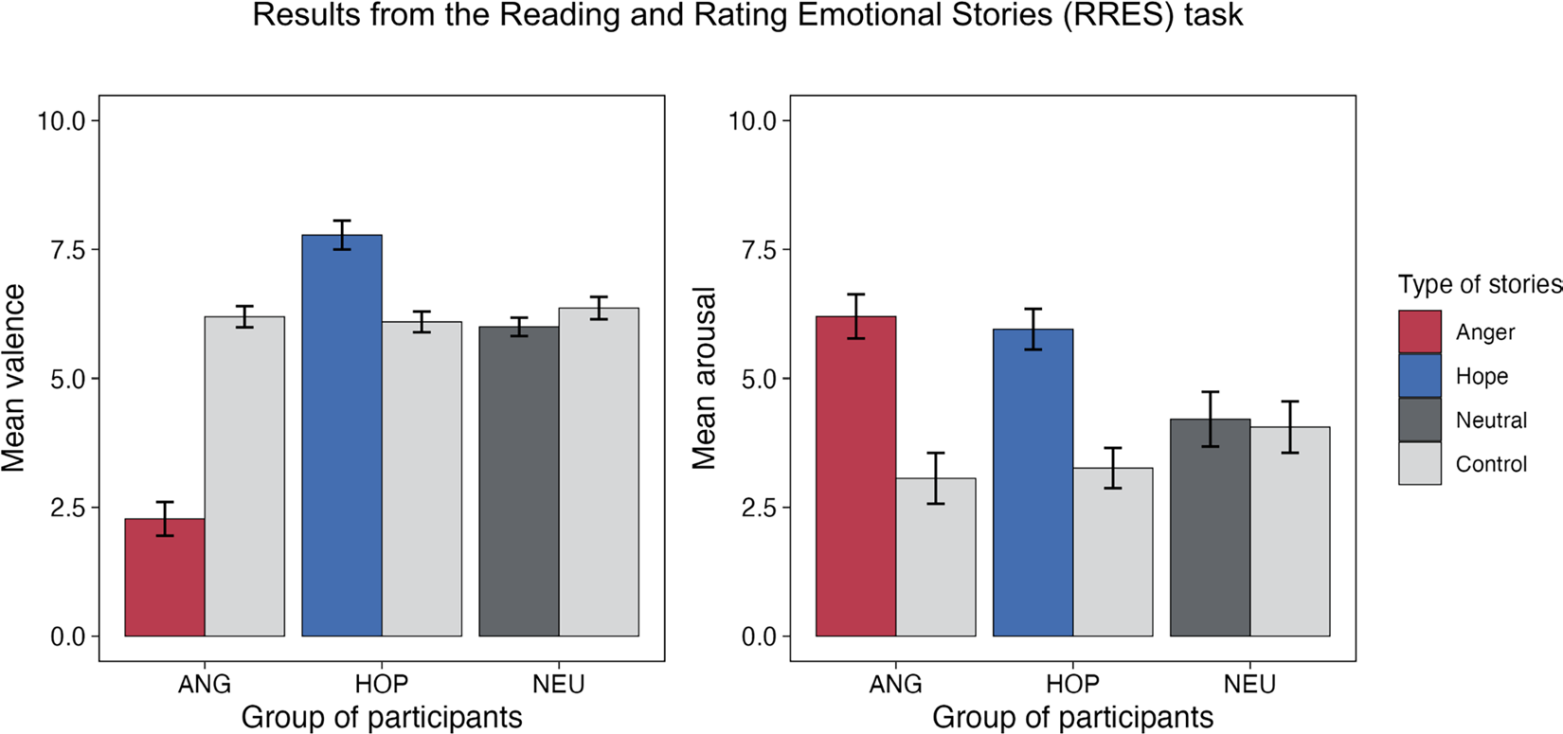
RRES validation. Mean arousal and valence ratings. Emotional stories are more arousing than neutral ones, anger stories are more negative, and hope stories - more positive. Note: Error bars represent 95% confidence intervals.

At the behavioural level, we checked the effectiveness of emotion elicitation in RRES by comparing the story ratings within and between groups. We demonstrate that the ECCS stories are effective in evoking emotional reactions, as indicated by the expected differences in valence and arousal (Fig. 6). A repeated-measures ANOVA showed a significant interaction effect between group and story type on arousal, F(2,157) = 56.02, p < .0001, η²p = 0.42. Post hoc tests confirmed that neutral stories were less arousing than both anger (mean difference: −1.99, p < 0.001) and hope (mean difference: −1.74, p < 0.001), while anger and hope stories did not differ in terms of arousal (mean difference: 0.25, p = 1.00). As expected, we did not observe significant differences in arousal ratings between target and control stories in the NEU group (mean difference 0.15, p = 1.00). For valence, a significant interaction effect between the group and story type was also found, F(2,157) = 335.69, p < .001, η^2^p = 0.81. Hope stories were rated more positively than neutral (mean difference: 1.78, p < .001) and anger (mean difference: 5.50, p < .001), while anger was rated most negatively (anger - neutral mean difference −3.72). We did not observe significant differences in valence between target and control stories in the NEU group (mean difference: −0.36, p = 0.13).

To validate the RRES task at the brain level we performed whole-brain analyses using a general linear model in SPM12. Timing corresponding to each condition (target story, control story, and ratings), along with multiple confounds (6 realignment parameters: 3 translations and 3 rotations, framewise displacement [FD] as a summary metric, and motion-censored volumes based on FD threshold > 0.5 mm) were modelled at the subject level. The hemodynamic response was modelled with a canonical response function as implemented in SPM12. Data was filtered with a 128 Hz high-pass filter. At the subject level we estimated contrasts corresponding to brain activation while reading target stories relative to passive rest^58^. At the group level we performed one-sample t-test, separately for each group.

**Fig. 7:**
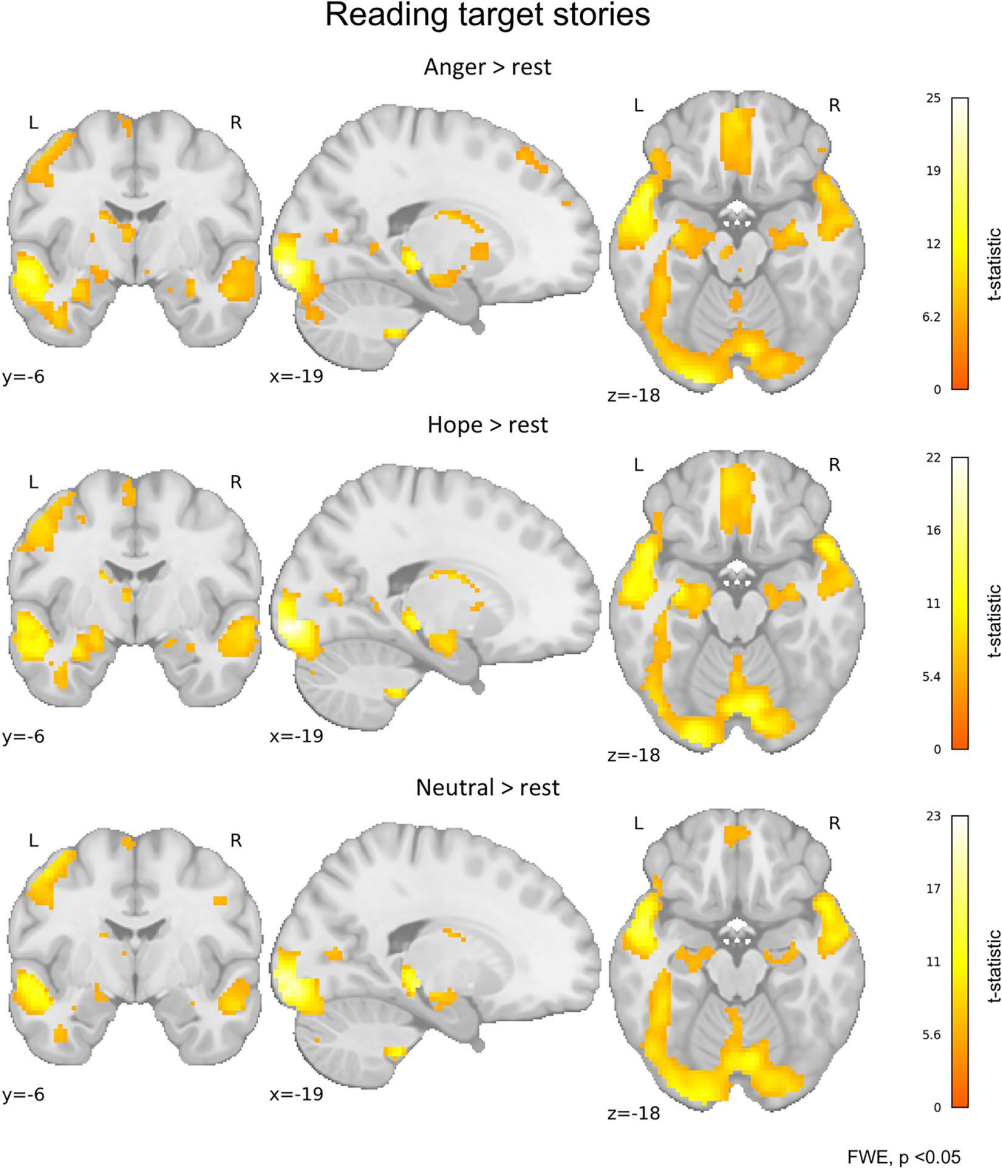
Whole-brain activations during target story reading (one-sample t-tests in each group). Respective panels demonstrate the results for each group of participants: ANG (n = 54), HOP (n = 52), and NEU (n = 48). The colour bars represent t-statistic ranges. All maps are thresholded at p < 0.05, FWE (Family-Wise Error) corrected at the voxel level.

In RRES, we decided to report brain activity during story reading relative to rest because our primary goal was to demonstrate regions broadly involved in reading and emotion processing. As expected, we observed increased involvement of brain areas within the reading network, including the ventral occipitotemporal cortex, inferior frontal gyrus, and superior and middle temporal gyri^59,60^. We also observed engagement of the limbic system, including brain areas such as the hippocampus, cingulate gyrus and insula^61,62^ (Fig. 7).

#### CET Validation

**Fig. 8:**
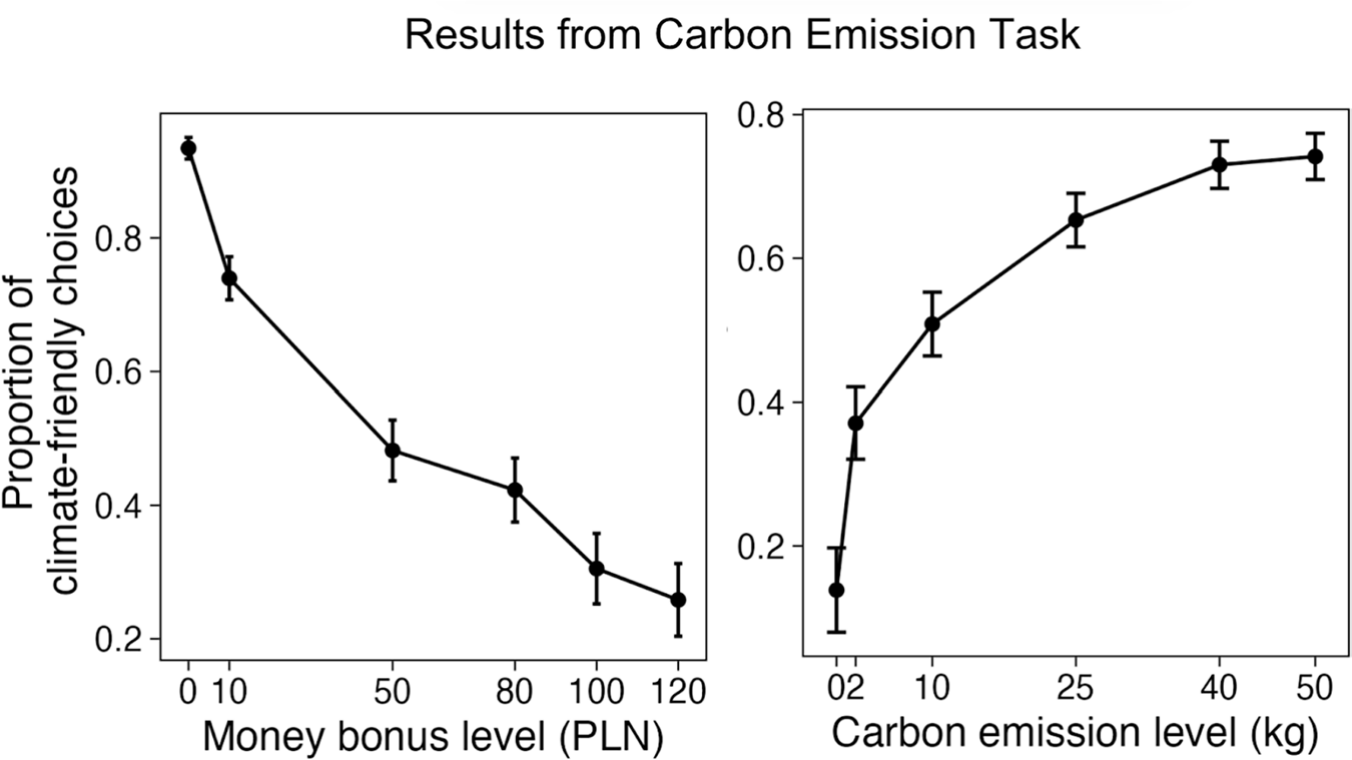
The proportion of climate-friendly choices depends on the size of the monetary bonus, as well as on how much CO_2_ emission can be reduced. Error bars indicate 95% confidence intervals.

To demonstrate the validity of the CET task, we showed that the proportion of climate-friendly choices linearly decreases with the size of the monetary bonus, and linearly increases with the amount of CO_2_ emission reduction (Fig. 8). Furthermore, we analyzed the responses to 10 “rational trials” and 12 “dummy trials”. In the rational trials, 144 participants (90%) consistently selected the rational option, such as choosing 100 PLN over reduction of 0 kg CO_2_. Among the remaining participants, 10 (6.3%) answered rationally in 9 out of 10 trials. In the dummy trials, 139 participants (86.8%) answered correctly in all 12 trials. Among the remaining participants 17 participants (10.6%) answered correctly in 11 out of 12 trials.

To test the validity of the CET task at the brain level we performed a whole-brain analysis using a general linear model in SPM12. Timing corresponding to each event, along with multiple confounds (6 realignment parameters: 3 translations and 3 rotations, framewise displacement [FD] as a summary metric, and motion-censored volumes based on FD threshold > 0.5 mm) were modelled at the subject level. The hemodynamic response was modelled with a canonical response function built into SPM12. Data was filtered with a 128 Hz high-pass filter. First-level contrasts comparing brain activation during climate-related decision-making target trials and dummy trials were entered into the second-level one-sample t-test model (n = 154).

**Fig. 9:**
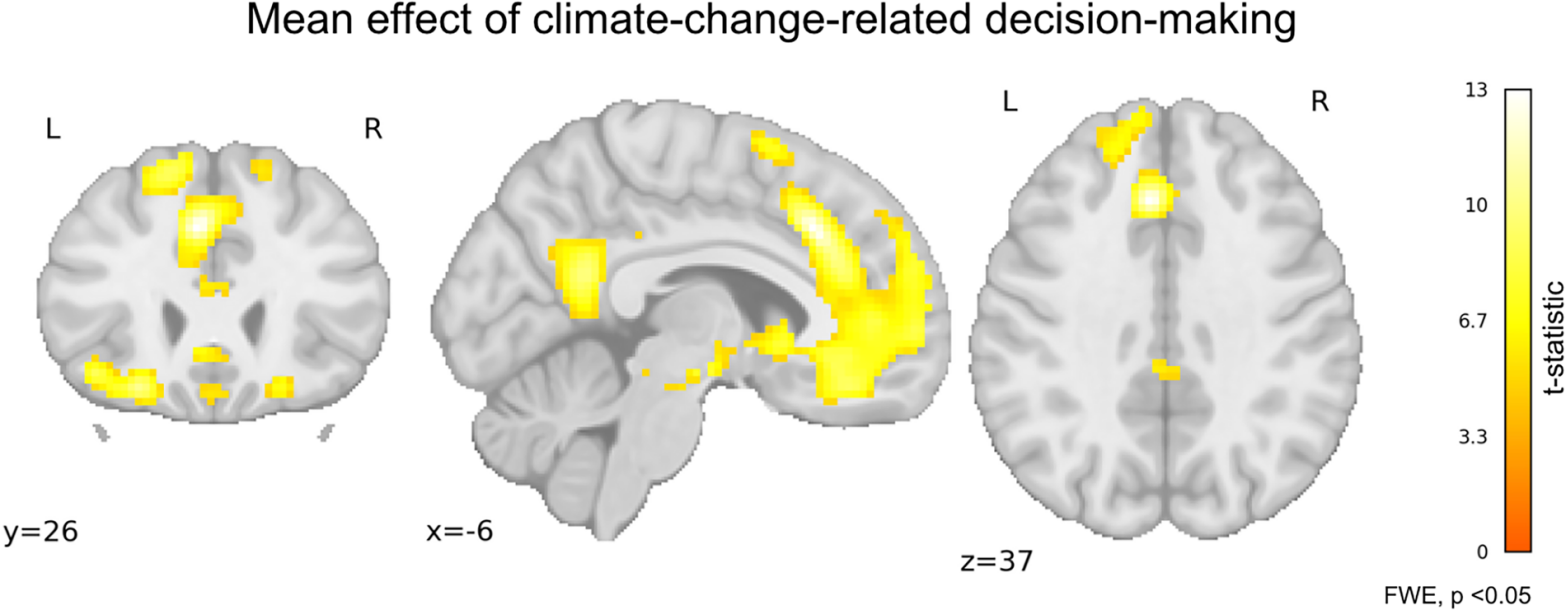
Regions of increased activity in climate-change-related decision-making (N = 154). Statistical maps represent the contrasts for target trials compared to dummy trials (one sample t-test). The colour bar represents the t-statistic range. All maps are thresholded at p < 0.05, FWE (Family-Wise Error) corrected at the voxel level.

In CET, we wanted to confirm the activation in areas related to decision-making and valuation of the worth of a particular outcome (monetary bonus and CO_2_ reduction). For this purpose, the CET > dummy contrast was an appropriate choice. We observed increased activation in brain areas typically associated with decision-making, such as the superior and middle frontal gyrus, ventromedial prefrontal cortex, precuneus, hippocampus, posterior cingulate cortex, insula, and orbitofrontal cortex^63–65^ (Fig. 9).

## Supplementary information

Supplementary Information

## Data Availability

All analysis code is available at https://github.com/nencki-lobi/climate-brain.
