# Supplementary material for: CLIMATE BRAIN - Questionnaires, Tasks and the Neuroimaging Dataset
Source: Sci Data. 2025 May 1;12:726. doi: 10.1038/s41597-025-05038-0 (PMC12045999; doi:10.1038/s41597-025-05038-0)
Supplement: Supplementary file 1 — Supplementary Information [file 41597_2025_5038_MOESM1_ESM.pdf]

Experimenter Checklist

Participant code (sub - _ _ _ _ _)

If anything goes wrong, note it next to the relevant checklist item.

Researchers initials

Participant coordinator: _ _ Scanner room personnel: _ _

Participant coordinator:

- ☐ Provide study information
- ☐ Obtain written consent for participation
- ☐ Start a demographic and questionnaire procedure on the computer
- ☐ Start the MRI safety survey: Enter correct participant code
- ☐ Hand the MRI safety survey over to the scanner room personnel
- ☐ Demonstrate MRI response pad usage (right hand, buttons for fingers 2 and 3)

Scanner room personnel:

- ☐ Review MRI safety survey and approve/deny participation
- ☐ Instruct participants to remove metal objects, take off glasses
- ☐ Launch `neurogrieg.exp` → Enter the participant code ()
- ☐ **Audio check:** "Can you hear me?"
- ☐ Start the localizer run
- ☐ **Training session and T1:** Launch `stories_training.sce`
    - ☐ First screen: "Can you see the text clearly?" If yes, proceed. If no, adjust (scanner compatible glasses)
    - ☐ "Do you understand the instructions?" → If yes, proceed.
    - ☐ Inform that an anatomical scan will be performed in the meantime.
    - ☐ Start T1, fieldmap
- ☐ RRES Task:
    - ☐ `stories_R1.sce` → Check condition: ANG/HOP/NEU
    - ☐ `stories_R2.sce` → When finished, check participants' wellness
    - ☐ `stories_R3.sce` → When finished, check participants' wellness
- ☐ CET Task**:**
    - ☐ `cet_training.sce` → "Do you understand the instructions?" → If yes, proceed to comprehension check questions. Correct answers.
    - ☐ `cet.sce`

Participant coordinator:

- ☐ Debriefing
- ☐ Payment

RRES Instruction

Note: The original instructions were presented in Polish. The original version is available on the GitHub repository.

In a moment, you will read several stories describing various situations. All stories are based on real events and were created from the accounts of people who agreed to share their experiences with us.

Your task will be to read each story and then assess the intensity of your own emotions using the scales described below:

1. Do the situations described evoke rather negative or positive emotions in you?
2. To what extent do the situations described emotionally arouse you? Do you feel a complete lack of arousal, or strong arousal, agitation, or excitement?

Pay particular attention to the emotions you feel while reading the stories.

You will provide your answers using two buttons located on the response pad. The left button will be used to change the answer to the left, and the right button to change it to the right.

You will always have the same amount of time to respond: 5 seconds for each question. After this time, your answer will be recorded, and you will move on to the next screen.

When responding, try to use the full range of the rating scale. There are no right or wrong answers; respond according to your first impression. You will start with a short training session.

RRES Guide for the Experimenter

Initial Voice Instructions

I will now display the instructions for the first task. You can move to the next screen using any button.

FAQ

What do the scales represent?

On the first scale, you will rate whether your emotions are generally positive or negative, regardless of their intensity. On the second scale, you will rate how aroused or emotionally reactive you feel, indicating the strength of your emotional response.

ECCS stories used in the RRES task

Table S1. English translation of Emotional Climate Change Stories that were used in the RRES task. Polish version of the stories can be accessed through the related OSF project and the GitHub repository.

Group	Run	Story	Code
ANG	r1	For several decades governments of many countries knew what the global climate would look like if greenhouse gas emissions were not reduced. Nevertheless, the short-term interests of the rich were more important than the well-being of future generations.	ANG21
ANG	r1	Alicia is the head of a large steel manufacturing company. She had an opportunity to obtain funds to introduce environment-friendly solutions at her company, but after some calculations, she decided not to apply for the subsidy. She concluded that the investment would not pay off, even though her company's environmental impact would be reduced.	ANG12
ANG	r1	A certain politician downplayed reports about the threat of drought caused by climate change. When the town suddenly ran out of water, he sneaked off to his lakeside summer residence to avoid journalists.	ANG3
ANG	r1	August is a senator and his wife works at a state-owned company responsible for heavy environmental pollution. August blocks financial support for climate action to protect his wife's financial interests.	ANG20
ANG	r2	In a recent media interview, a celebrity criticized people who use plastic shopping bags. The same person travels by private jet several times a week and invests in crude oil extraction.	ANG8
ANG	r2	Philip spends a lot of time browsing social media. He has just seen a photo of one of the richest people on earth. The satisfied billionaire hugs a koala in front of his private jet. The caption under the picture reads: “It would be sufficient if each of us did a small thing for the planet.”	ANG30
ANG	r2	Once a week Frank buys groceries at a large supermarket. Each time he asks the cashier for new plastic shopping bags. When he brings his shopping home, he throws the bags away without a second thought.	ANG9
ANG	r2	A corporation commissioned a report on its environmental impact. As the report turned out to embarrass the corporation, the management decided to conceal it. Employees were forbidden to disclose any information on this subject.	ANG6
ANG	r3	Lisha is very rich and likes to change her wardrobe	ANG25

		frequently. Ever since she has discovered a website with very cheap clothes, she orders 20-30 items a week. Some turn out to be a poor fit, so she throws them straight into the garbage bin. Still, Lisha does not consider this wasteful, because the clothes were ridiculously cheap.	
ANG	r3	The editors of a weekly magazine found out about a new, high-profile study proving that burning coal has a much greater negative impact on the environment than previously thought. However, since the magazine is sponsored by a major energy producer, journalists working there were forbidden to publish any stories about this topic.	ANG7
ANG	r3	Agatha is watching a TV documentary about climate change. She does not like what she sees and believes that the film spreads disinformation. “People in our region used to burn coal and there was never any smog. It is only now that they've invented this smog.“, thinks Agatha.	ANG1
ANG	r3	The city council is debating how to spend the budget reserved for transport infrastructure. The choice is between constructing bicycle lanes or adding another car lane to the main road through the town. The councillors vote to build another car lane.	ANG17
CON	r1	Greg often studies in the library and uses the computer in the reading room. He usually sits at the computer next to the window, with his back to the door. Today, however, he is waiting for a friend, with whom he will be studying together, so he sits at another table, to be able to see when his friend enters the reading room. He turns on the computer and starts working.	NEU5
CON	r1	As he was slowly walking back home from work, Mark was thinking about the ingredients he needed to buy for his dinner when a passer-by pointed to his undone shoelace. Mark knelt down to tie it.	NEU3
CON	r1	Anne hasn't changed the recording on her voicemail for a long time. It occurred to her that she might make the greeting more professional. She recorded a new message and listened to it. She was pleased with what it sounded like, so she saved it and put the phone down.	NEU2
CON	r1	Riding a tram, Monica is reading an interesting book. As she turned the page, a lock of her hair fell to her shoulder, interfering with her reading. Still reading, Monica scratched her shoulder and tucked the strand behind her ear.	NEU22
CON	r2	Having watched the last episode of his favourite series, Olivier asked his friend if he could recommend another one. Olivier got several recommendations, read the reviews and chose one title. The first episode turned out to be very interesting, so he intends to keep watching it.	NEU16

CON	r2	Susan likes to go for a walk around her neighbourhood in the evening. When she comes to the roundabout, she usually turns right and walks towards the river. On the river bank, she often meets her neighbours with dogs. This time she turned left and walked to the lake. “I rarely visit this area, maybe I'll do it more often.”, she thought.	NEU26
CON	r2	George decided to take a break from work and have some lunch. He usually eats a bun, but this time he only had some bread at home. He cut two slices, took the rest of the ingredients out of the refrigerator, and made sandwiches. He took the plate with the sandwiches to his desk and ate them there as he browsed a news website.	NEU25
CON	r2	Monica entered the room, opened the wardrobe and bent down to reach into one of the lower shelves. She took out her brown pants and put them on. Then she opened a drawer in her dresser and took out a belt. She passed it through the belt loops on her pants and fastened the buckle.	NEU4
CON	r3	Alex came to class a few minutes early and had nothing to do. She noticed that the blackboard had not been erased after the previous lesson. She took the sponge and wiped it. Then she sat down, took her notebook out of her bag, and waited patiently for the class to begin.	NEU17
CON	r3	Simon is examining some forms he has to fill in to enrol his daughter in kindergarten. He picks up a pen and writes his first and last name in the first field. Then he enters his child's first and last name. He gives his phone number and puts a checkmark next to a personal data processing consent clause.	NEU27
CON	r3	Milo is driving a car with his wife and children in passenger seats. It is quiet – the children are sleeping in the back seats, and his wife calmly watches the road. A slight drizzle has just started. Milo turns on the wipers to clean the windshield and improve visibility.	NEU10
CON	r3	Malik went to the cinema. As he was leaving after the film, he saw his friend on the other side of the hall. He waved his hand at her, and she waved back. He passed between rows of chairs and walked down the steps on the opposite side to say hello to his friend and ask her how she liked the film.	NEU18
HOP	r1	Christiana is a diplomat. Thanks to her hard work, the governments of the entire continent came to an agreement and signed a pledge to limit climate change. She knows she still has a lot of work to do, but she is proud to be part of this groundbreaking endeavour.	HOP22
HOP	r1	Daria noticed that more and more vegetable substitutes for meat and dairy products were available at the local store. She mentioned it to her friend Wiktoria, who realized that the same happened at her favourite store.	HOP19

	r2	Since then, the women have discovered that they both like a plant-based diet and have been swapping favourite vegetarian recipes.	
HOP	r1	Since last year, a certain country has been absorbing more carbon dioxide than it generates. Despite many radical changes and restrictions that were required for that to happen, residents report that their quality of life has actually improved. This shows that systemic solutions are effective in combating climate change.	HOP7
HOP	r1	Esther's daughter told her mother that the management of her school was considering the installation of solar panels. Hearing that, Esther asked other members of the Parents Council to support the director's initiative. One parent is an accountant, and he volunteered to help process the purchase of panels for the school, free of charge.	HOP15
HOP	r2	Amara is an engineer and she specialises in fire safety. She has invented a special coating for buildings to protect them from fires, which are increasingly common. What is more, the coating is made of industrial waste, which would normally end up in a landfill.	HOP23
HOP	r2	Penelope is a city bus driver. She recently had an opportunity to drive an electric bus and liked it very much. She is now urging her colleagues to petition the company to introduce more electric vehicles on all lines in the city.	HOP13
HOP	r2	Thanks to local community pressure, the authorities in your area have obtained permission to build a renewable energy power plant to replace the former coal-fired plant. These steps will contribute to the mitigation of the negative effects of climate change.	HOP5
HOP	r2	Julian is working on a technology to capture carbon dioxide from industrial stacks. His invention – which is many times more effective than any solution currently in use – will soon be available on the market. Julian does not intend to apply for a patent, because he wants the technology to be cheap and widely available.	HOP26
HOP	r3	Isabella used to commute to work by car. When she switched to a bicycle, she had to get used to the new way of transportation. Today Isabella would not want to switch back to the car – she likes the opportunity to exercise in the morning, while avoiding traffic jams and at the same time making a contribution to climate protection. She has also convinced a few colleagues to switch to the bike.	HOP17
HOP	r3	Christian and Harper are protesting outside the Town Hall, demanding that the burning of low-quality fuel in stoves be banned. Thanks to their protest, the authorities impose the ban and decide to allocate additional	HOP16

		subsidies for the replacement of stoves in the homes of residents who cannot afford it on their own.	
HOP	r3	Emily watched an inspiring lecture on possible solutions to the problem of climate change. She then published a post about it on social media and sent a recording of it to her friends. Emily always thought that hardly anyone among her acquaintances was interested in the climate, but it turns out that the post has been viewed and commented on by hundreds of people.	HOP11
HOP	r3	Yoshida has discovered a new species of bacteria. The bacteria are able to break down plastic that would otherwise be deposited in landfills. Yoshida's discovery gained so much publicity that he was awarded another research grant. There are reasons to hope that his solution can be implemented on a large scale.	HOP27
NEU	r1	Janine is planning a budget for a business trip next week. She has already checked how much she will pay for accommodation, transport and lunches in the city. She had estimated the costs of the trip earlier, but now she must give her boss the exact figure. She is using a calculator to make sure she has not made any mistakes.	NEU24
NEU	r1	Ivonne noticed that the light bulb in her bathroom was burned out. Luckily, she found a spare bulb in a drawer in the kitchen. She left the bathroom door ajar, so as to let in some light from the hall. She climbed a stool in the dark bathroom and changed the light bulb. Then she flipped the switch to test whether it worked.	NEU6
NEU	r1	Louis entered the bus and took a seat at the end of the vehicle. As he watches the changing landscape through the window, he thinks about his plans for the coming weekend. He notices a poster advertising an interesting exhibition. “Maybe I can still get tickets for this exhibition.”, he wonders.	NEU12
NEU	r1	Cornelia finished her morning workout around eight. She picked up her towel from the floor and drank some water from her bottle. At first, she was surprised to find the water tasted of lemon. Then she remembered adding some lemon juice to the water in the morning to add some vitamins. When she finished drinking, she put the bottle back in her bag and headed for the changing room.	NEU30
NEU	r2	Jeremy is preparing a grocery shopping list. He looks in the refrigerator to check what he needs to buy. Then he knocks on the door of his son's room to ask if he wants to add anything to the list.	NEU14
NEU	r2	Angela turned off the TV and yawned. It was late and she felt like going to bed. She changed into her pyjamas and set the alarm clock. “Tomorrow I have to get up earlier than yesterday because I need to post a package before I go to work.”, she remembered and reset the alarm clock	NEU15

		fifteen minutes earlier.	
NEU	r2	Stan is cooking pasta in the kitchen. He walks around barefoot, but after a while he notices that his feet have become cold. “I have to wait for the water to boil anyway, so I might as well put on socks.”, he thought. He goes to his room, takes the socks out of the drawer and puts them on. When he is back in the kitchen, the water is already boiling. He tosses the pasta into the water.	NEU29
NEU	r2	Adam is expecting guests. He entered the bathroom to check if there was soap in the dispenser and noticed that there was no towel. “Do I have any clean towels?” he thought as he left the bathroom to look for a towel in the closet. He took a small beige towel and returned to the bathroom to hang it on the hook next to the sink.	NEU21
NEU	r3	John entered the conference room and sat down on a chair. Only then did he look around and think, “If the front row seats have not all been taken yet, I'll switch.” He got up and moved closer to the screen at the front of the room.	NEU1
NEU	r3	Margaret printed some documents she needs to submit to the municipal office. She arranges the pages in the correct order and staples them with a stapler. She then stuffs them into a folder and puts the folder in her bag. “I'm glad this is now off my mind.”, she thinks.	NEU11
NEU	r3	Billie returned home after a long day of work in the field. He wanted to relax in his favourite armchair, but he noticed that it was still cluttered with the grocery bags he had left there in the morning. “I'll unpack them later, I can rest on the couch.”, he thought. As he lay down on the couch, he continued to think about his chores for a while, but soon fell asleep.	NEU23
NEU	r3	Mary has just returned home from a trip. She hung up her coat, put down the keys, and checked her watch. Then she went into the kitchen to get a glass of water and realized that she did not remember what time it was. Sipping the water, she moved closer to the kitchen clock and checked the time again.	NEU9

CET Instruction

Note: The original instructions were presented in Polish. The original version is available on the related GitHub repository.

Carbon dioxide (CO_2) is considered a key factor contributing to climate change. Scientists around the world agree that climate change can only be mitigated if CO_2 emissions are significantly reduced.

To limit CO_2 emissions, a market for trading CO_2 emission allowances was created. Factories or power plants must purchase allowances, called emission certificates if they want the right to emit CO_2. The number of these certificates is limited.

For this study, we have purchased a certain number of CO_2 emission certificates from the market. Some of these certificates will be destroyed (permanently removed from the market). How many certificates are destroyed will depend on your decisions in the task.

If a CO_2 emission certificate is destroyed, no company will be able to emit that amount of CO_2. This will result in less CO_2 being released into the atmosphere, contributing to climate change mitigation.

In this task, you will be asked to make 48 decisions that could either reduce CO_2 emissions or increase your compensation.

To help you understand the consequences of your choices, we will additionally represent CO_2 emissions as the number of kilometres driven by an average car.

In each of the 48 trials, you will be asked to choose one of two options. You can either take a financial bonus ranging from 0 to 120 PLN or remove a certificate allowing the emission of 0 to 50 kg of CO_2.

In each trial, you will have 10 seconds to make a decision. If you do not decide within 10 seconds, the screen will automatically move to the next trial.

If you do not make a decision, you will not receive any money, and no CO_2 will be removed. Not making a decision is disadvantageous for both you and the climate.

Your choices do not accumulate. We will randomly select one of your decisions, based on which you will either receive a bonus or a CO_2 emission certificate will be destroyed. Depending on your decisions and the result of the draw: You could receive up to 120 PLN or remove up to 50 kg of CO_2, equivalent to driving 250 km by car.

Choosing to remove CO_2 increases the chances of destroying a CO_2 emission certificate, which helps mitigate climate change. Choosing a financial bonus increases your chances of receiving a payout. To check if you are paying attention to the task, sometimes we will ask you to choose a specific option, “Choose the option on the left” or “Choose the option on the right.” This instruction will appear at the top of the screen. You will not receive a bonus for these decisions, and they will not involve any CO_2 emissions. These decisions will not be included in the draw.

You can select the option on the left using the left button, and the option on the right using the right button.

CET Guide for the Experimenter

Initial Voice Instructions

This task includes detailed instructions. You can navigate through them by pressing the left and right buttons.
Once you reach the final screen, I will ask if everything is clear and address any questions you may have.

Comprehension Questions

1. **What will you be choosing between?**
   Accepted answers: money/remuneration/benefit for me and carbon dioxide/emissions/certificates/benefit for climate/environment
2. **How will your final remuneration be determined?**
   Accepted answers: it is not cumulative/they don't sum up + it is chosen randomly/it is drawn from all of my choices
3. **What should you do when you see a choice between 0 PLN and 0 kg CO2?**
   Accepted answers: follow the instructions at the top, choose left or right depending on the instruction
4. **What happens if you don't choose any of the options within 10 seconds?**
   Accepted answers: neither I nor the climate benefits/I don't receive money, and I don't reduce CO2 + the program shows the next choice

FAQ

1. **How much does a single CO2 certificate cost?**

I cannot provide such information at this time. Please focus on making your decision based on the information available about the amount of the money bonus, the kilograms of CO2 and the kilometres driven in the car.

Tutorial corrective messages

1. **Wrong choice in the dummy trial.**
   Please pay attention to the instruction at the top of the screen—if it says 'choose the left option,' select the option on the left.
2. **Clicking multiple times during the same trial.**
   Did you try to change your answer? Unfortunately, only the first response counts, so please make your decision carefully.
3. **No decision within 10 seconds.**
   If you don't choose an option, that decision will still be included in the final draw, and neither you nor the climate will benefit.
4. **Irrational choice in the c0_m2 trial2 (participant selects 0 kg carbon reduction, instead of 50 PLN)**
   What was behind your choice in this trial? See, you could get a chance to get 50 PLN, but there was no risk that you will cause CO2 emissions. Is that clear to you?

Debriefing

We would like to inform you that in the last task, we had to tell you that the amount of compensation and emissions would be determined randomly to ensure the success of our experiment. However, in reality, we will make sure that neither you nor the climate will suffer any loss.

You will receive compensation of 80 PLN, making a total of 200 PLN for participating in the entire study. Additionally, on your behalf, we will destroy emission certificates equivalent to 25 kg of CO_2, which corresponds to the emissions from driving 125 km by car.

Full list of demographic and psychometric questionnaires

Note: The original questions were presented in Polish. The original version is available on the related OSF Project and the GitHub repository.

Demographic survey

---

1. Indicate your sex.

- Female
- Male
- Other (Please specify: _______)

---

2. Indicate your age.

- (Input field)

---

3. Which of the following best describes the area you live in?

- Big city
- Suburbs of a big city
- Small city
- Country village
- A farm or home in the countryside

---

4. What is your educational attainment?

- Incomplete primary
- Primary
- Lower secondary
- Vocational
- Upper secondary
- Bachelor’s degree or engineering degree
- Master’s degree or medical degree
- Doctorate (Ph.D.), habilitation, or professorship
- Other (Please specify: _______)

---

5. How concerned are you about climate change?

- Not at all concerned

- Not very concerned
- Somewhat concerned
- Very concerned
- Extremely concerned

---

6. What actions do you consider most important in addressing climate change?

- Collective actions (by states, institutions, corporations) [1]
- Individual actions (by ordinary people) [5]

(Scale from 1 to 5 where 1 represents collective actions and 5 represents individual actions)

---

7. Compared to other people, do you consider your lifestyle ...?

- Definitely climate-unfriendly [1]
- Definitely climate-friendly [5]

(Scale from 1 to 5 where 1 represents climate-unfriendly and 5 represents climate-friendly)

---

8. Do you have a driver's license?

- Yes
- No

---

9. How often do you use a car (e.g., as a driver or a passenger)?

- Once a year or several times a year
- Once a month or several times a month
- Once a week or several times a week
- Every day

---

10. Do you have any political views?

- No
- Yes
    - If yes: In politics, the terms /left/ and /right/ are used. Can you describe your political views using these terms?
        - No
        - Yes

---

11. Please indicate your political views.

(Visible only if the previous question's answers are both "Yes")

- Left [0]
- Right [10]

(Scale from 0 to 10 where 0 represents the left and 10 represents the right)

---

12. Which of the descriptions below comes closest to how you feel about your household income nowadays?

- Living comfortably on present income
- Coping on present income
- Finding it difficult on present income
- Finding it very difficult on present income

Perceived Climate Action Efficacy (PCAE)

Instructions: Please indicate your level of agreement with the following statements.

Response Options:

- 1 - Strongly disagree
- 2
- 3
- 4
- 5 - Strongly agree

I believe my actions can have a beneficial influence on climate change.
Actions I take personally can help reduce the impacts of climate change.
Climate change can be averted by mobilizing collective effort.
If we act collectively, we will be able to minimize the consequences of climate change.

Psychological Distance (PD) to climate change

Instructions: Please indicate to what extent you agree or disagree with the following statements. Use the scale provided below.

Response Options:

- 1 - Strongly disagree
- 2
- 3
- 4
- 5
- 6
- 7 - Strongly agree

My local area will be influenced by climate change.
It will be a long time before the consequences of climate change are felt.

Nature Relatedness (NR)

Instructions: For each of the following, please rate the extent to which you agree with each statement, using the scale from 1 to 5 as shown below. Please respond as you really feel, rather than how you think “most people” feel.

Response Options:

- 1 - Disagree strongly
- 2 - Disagree a little
- 3 - Neither agree or disagree
- 4 - Agree a little
- 5 - Agree strongly

My ideal vacation spot would be a remote, wilderness area.
I always think about how my actions affect the environment.
My connection to nature and the environment is a part of my spirituality.
I take notice of wildlife wherever I am.
My relationship to nature is an important part of who I am.
I feel very connected to all living things and the earth.

Inventory of Climate Emotions (ICE)

Preamble: Perhaps you have heard that the Earth is currently experiencing climate change. The aim of this questionnaire is to examine your feelings on this subject.

Instructions: Rate the extent to which the following statements apply to you. For each statement, select one answer on the scale from "strongly disagree" to "strongly agree".

This questionnaire is not intended to verify your knowledge, so there are no right or wrong answers. Choose the answer that best describes what you feel.

Response Options:

- Strongly disagree
- Somewhat disagree
- Neither agree, nor disagree
- Somewhat agree
- Strongly agree

**ANG14**	I feel angry that the political and economic system that we live in harms the climate.
**ANG13**	I am outraged that politicians allowed climate change to come this far.
**ANG10**	I feel outraged at corporations that harm the climate.
**ANG3**	I feel anger when I think of politicians who delay efforts to mitigate climate change.
**DIS5**	It annoys me to watch people succumb to climate hysteria.
**DIS7**	I am annoyed by the constant publicity around climate change.
**IND2**	I am bored of hearing about climate change.
**IND13**	I am surprised that people experience strong emotions in connection with climate change.
**EMP12**	The increasing public engagement with climate change gives me hope.

**HOPF9**	I believe that there are emerging solutions that will allow us to stop climate change.
**CHECK1**	To convince us that you are reading this, please, just mark the option “Strongly disagree”.
**HOPF8**	Concrete actions for the climate allow me to be optimistic about the future.
**EMP7**	Social mobilisation in the fight against climate change makes me feel that together we can achieve this goal.
**POWL11**	I feel confused about what I can do to reduce climate change.
**POWL7**	I am overwhelmed by how many aspects of life would need to be changed to limit climate change.
**POWL2**	As an individual, I feel powerless with little agency over what happens with the climate.
**POWL13**	I feel helpless when I think of how difficult it is to live in a climate-friendly way.
**GUI11**	I have a guilty conscience about not doing enough to mitigate climate change.
**CHECK2**	To convince us that you are reading this, please, just mark the option “Neither agree, nor disagree”.
**GUI6**	It upsets me that I have a big negative impact on the climate.
**GUI8**	I feel guilty that my lifestyle contributes to climate change.
**GUI12**	I am angry at myself for not doing enough to limit my negative impact on the climate.
**ISO4**	I feel like one of the few people who actually understand what climate change entails.
**CHECK3**	To convince us that you are reading this, please, just mark the option “Strongly agree”.
**ISO5**	I feel lonely because most of the people around me don’t care about climate change as much as I do.
**ISO8**	I feel lonely because it’s difficult to talk about my climate change concerns with other people.
**ISO12**	I feel alienated because society considers concern for climate change as something strange.
**APP7**	Thinking about climate change makes me fear for the future of our children.
**HOPL5**	I am overwhelmed by the awareness of the approaching climate disaster.

**HOPL11**	Everything seems uncertain because of climate change.
**APP14**	I fear how climate change will affect me and my loved ones.
**SOR13**	The thought of so many species going extinct under the pressure of climate change fills me with sorrow.
**SOR6**	The thought that the world I know is disappearing forever because of climate change makes me sad.
**SOR4**	I feel sorry about the possibilities we are losing forever because of climate change.
**SOR14**	I am sad that so many living creatures suffer because of climate change.

Supplementary Figures

Figure S1. Distribution of sex across studied groups.

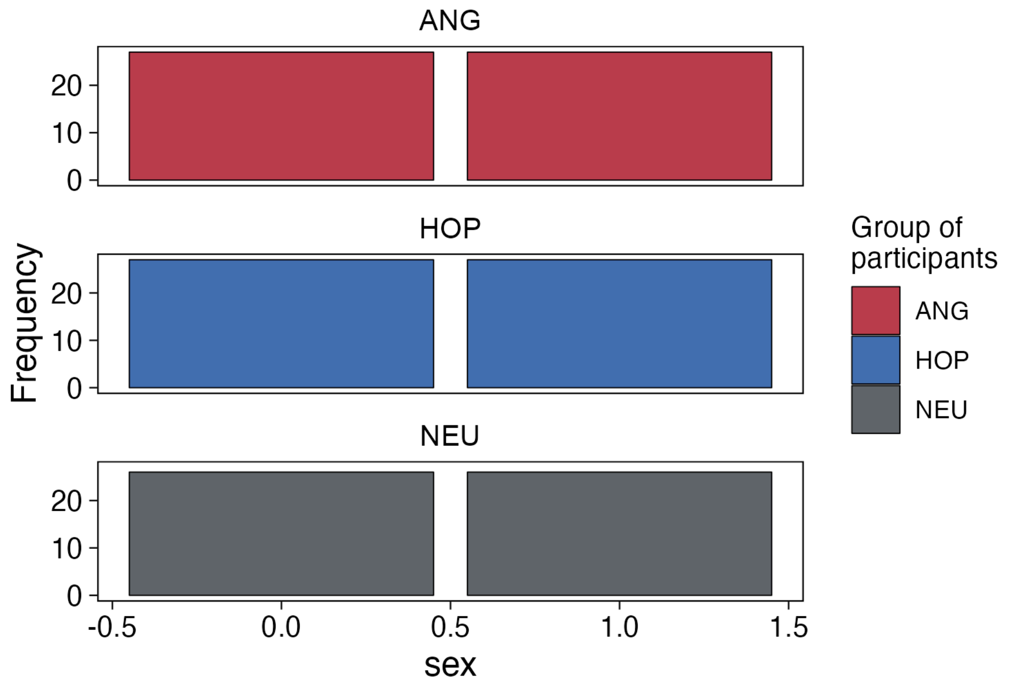

Sex - Levels: 0 = Female, 1 = Male

Figure S2. Distribution of age across studied groups.

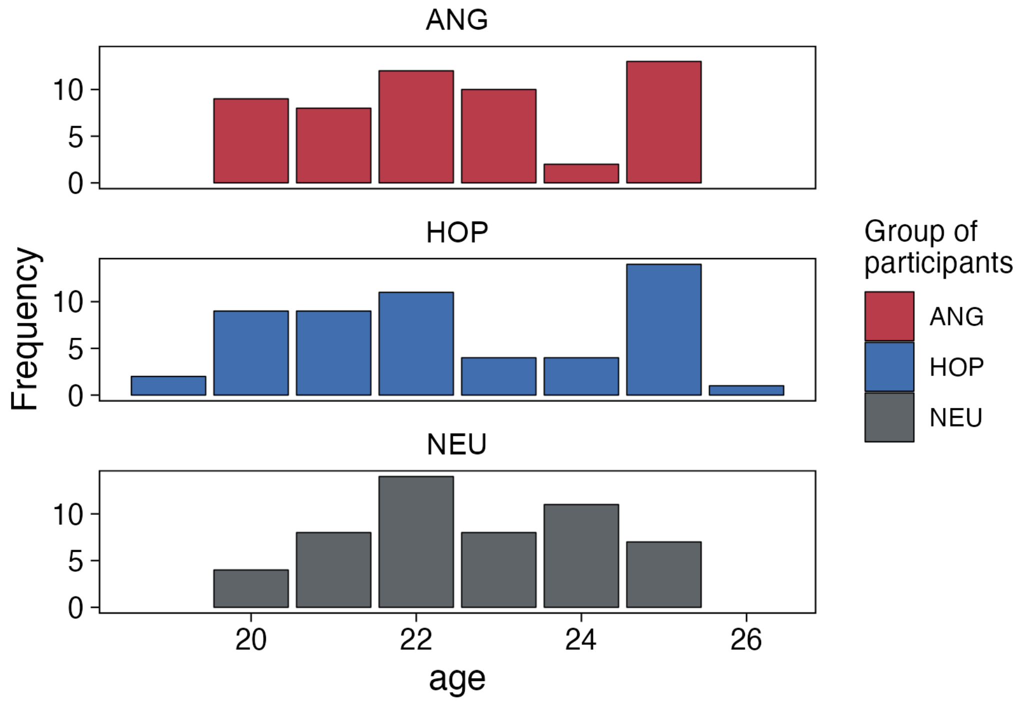

Age - Units: Years

Figure S3. Distribution of residence across studied groups.

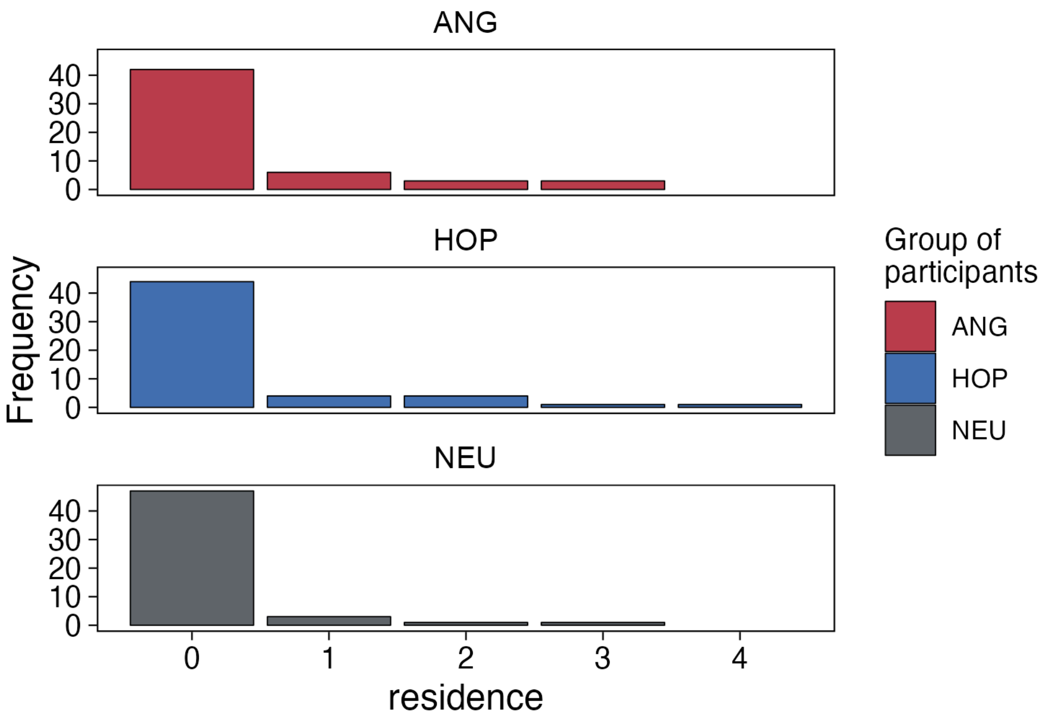

Place of residence - Levels: 0 = Big city, 1 = Suburbs of a big city, 2 = Small city, 3 = Country village, 4 = A farm or home in the countryside

Figure S4. Distribution of education across studied groups.

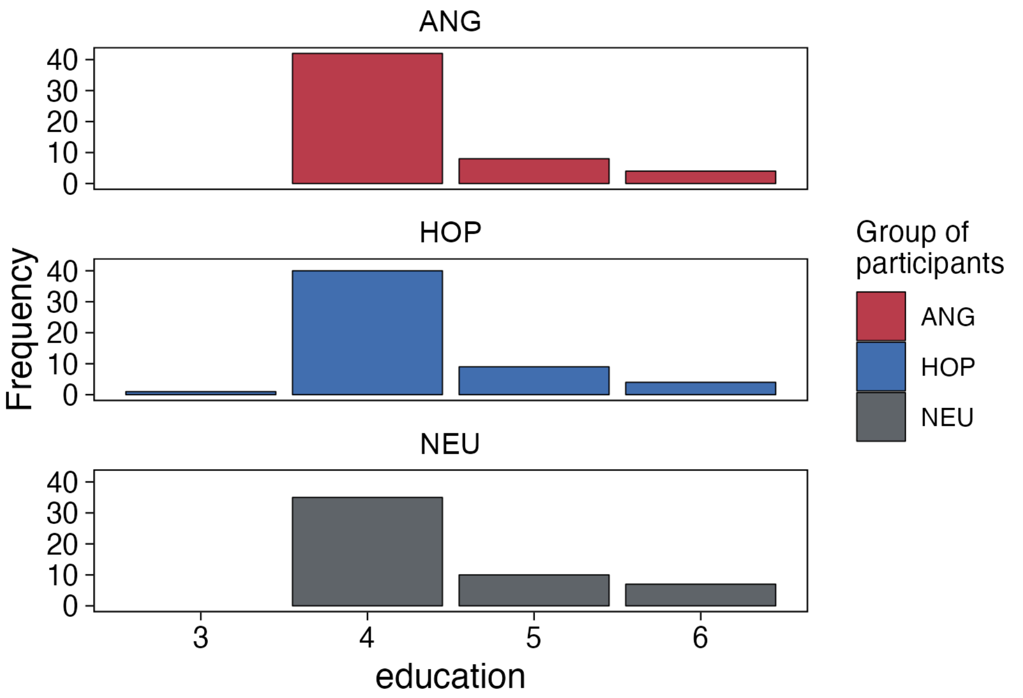

Education level - Levels: 0 = Incomplete primary, 1 = Primary, 2 = Lower secondary, 3 = Vocational, 4 = Upper secondary, 5 = Bachelor's degree or engineering degree, 6 = Master's degree or medical degree, 7 = Doctorate (Ph.D.), habilitation, or professorship, 8 = Other

Figure S5. Distribution of climate change concern across studied groups.

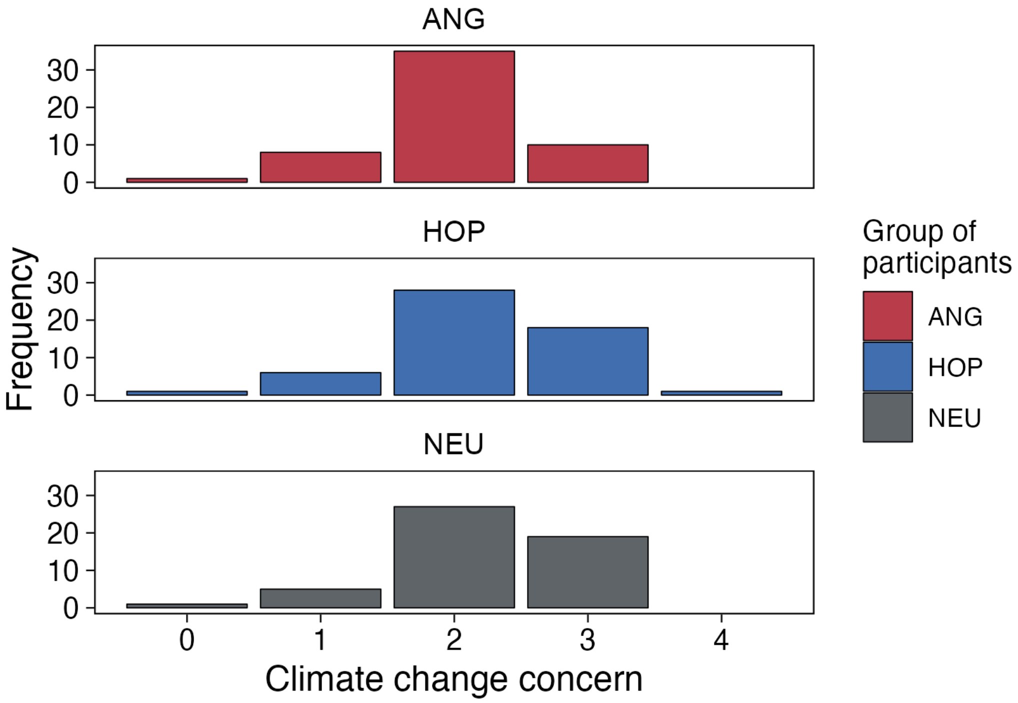

How concerned are you about climate change? - Levels: 0 = Not at all concerned, 1 = Not very concerned, 2 = Somewhat concerned, 3 = Very concerned, 4 = Extremely concerned

Figure S6. Distribution of perceived importance of climate action across studied groups.

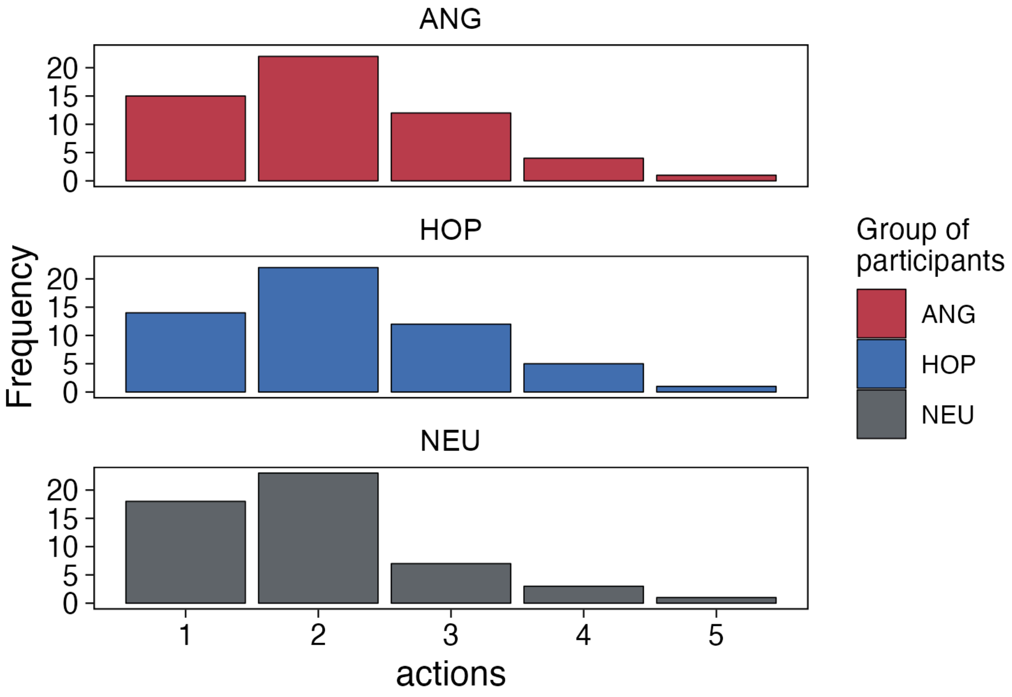

What actions do you consider most important in addressing climate change? - Units: Points on a scale, where: 1 - Collective actions (by states, institutions, corporations), 5 - Individual actions (by ordinary people)

Figure S7. Distribution of perceived climate friendliness across studied groups.

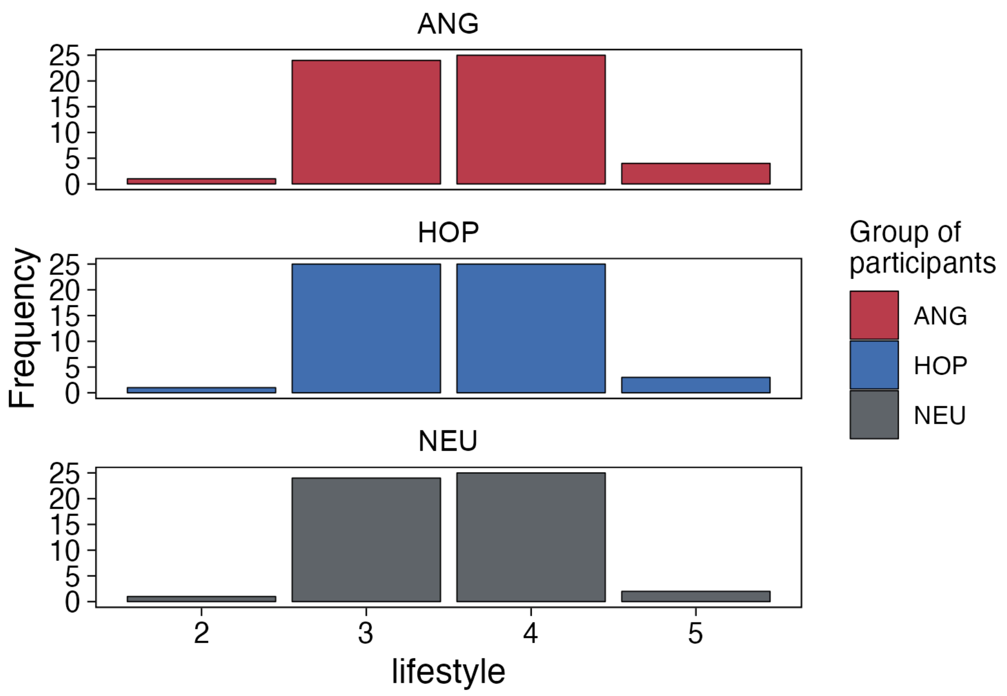

Compared to other people, do you consider your lifestyle ...? - Units: Points on a scale, where: 1 - Definitely climate-unfriendly, 5 - Definitely climate-friendly

Figure S8. Distribution of drivers' licence ownership across studied groups.

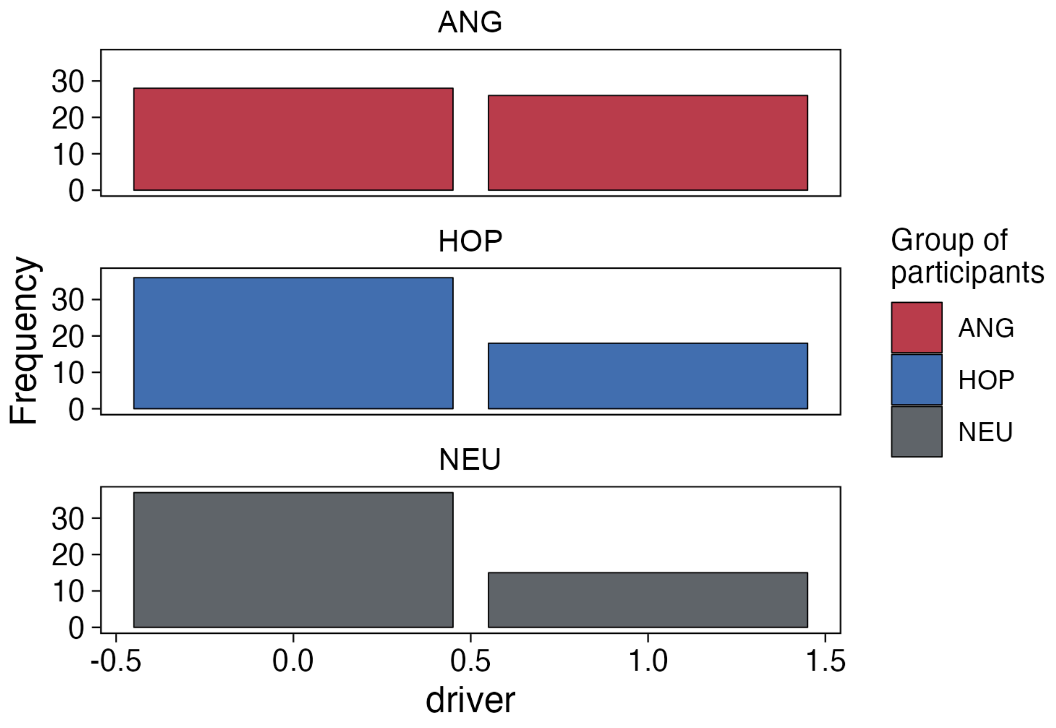

Do you have a driver's license? - Levels: 0 = Yes, 1 = No

Figure S9. Distribution of car usage frequency across studied groups.

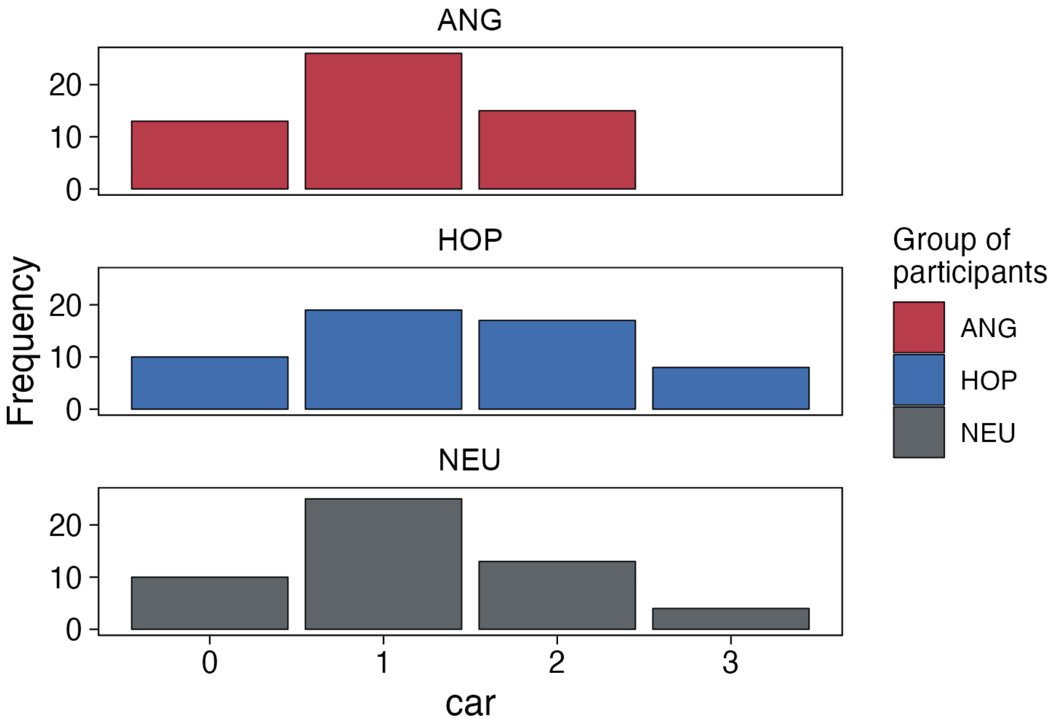

How often do you use a car (e.g. as a driver or a passenger)? - Levels: 0 = Once a year or several times a year, 1 = Once a month or several times a month, 2 = Once a week or several times a week, 3 = Every day

Figure S10. Distribution of political affiliation across studied groups.

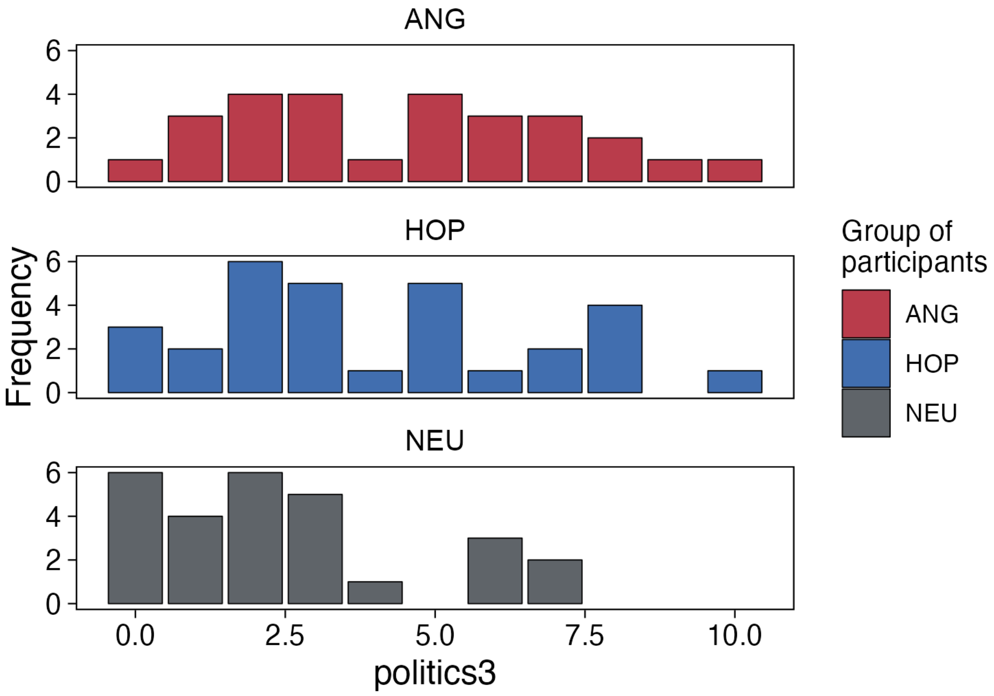

Please indicate your political views. - Units: Points on a scale, where: 0 - Left, 10 - Right

Figure S11. Distribution of socio-economic status across studied groups.

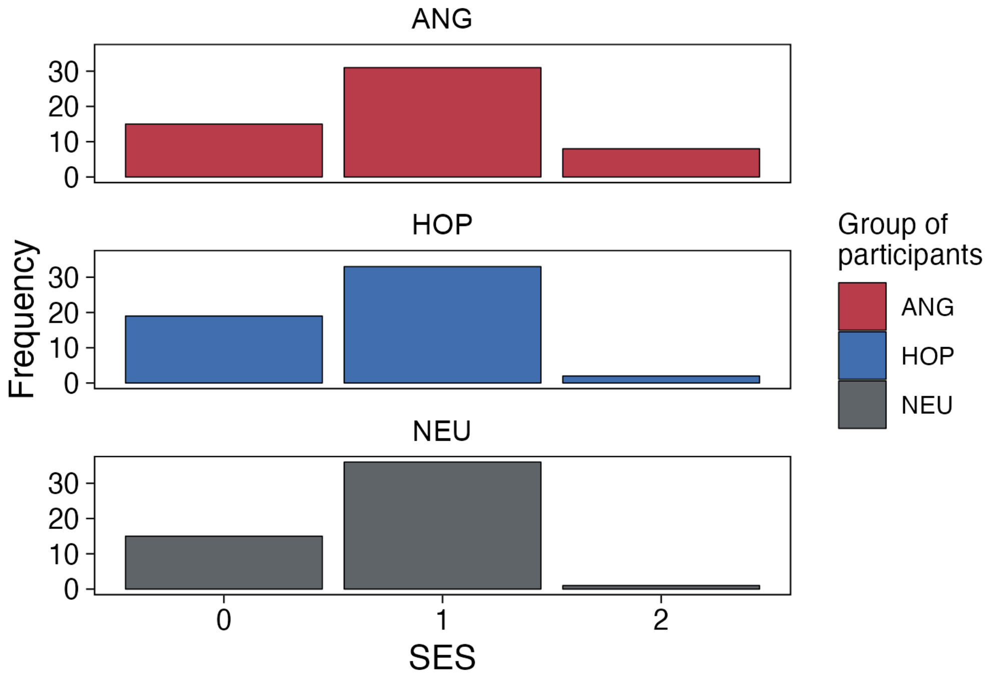

Which of the descriptions below comes closest to how you feel about your household income nowadays? - Levels: 0 = Living comfortably on present income, 1 = Coping on present income, 2 = Finding it difficult on present income, 3 = Finding it very difficult on present income

Figure S12. Distribution of perceived individual climate action efficacy.

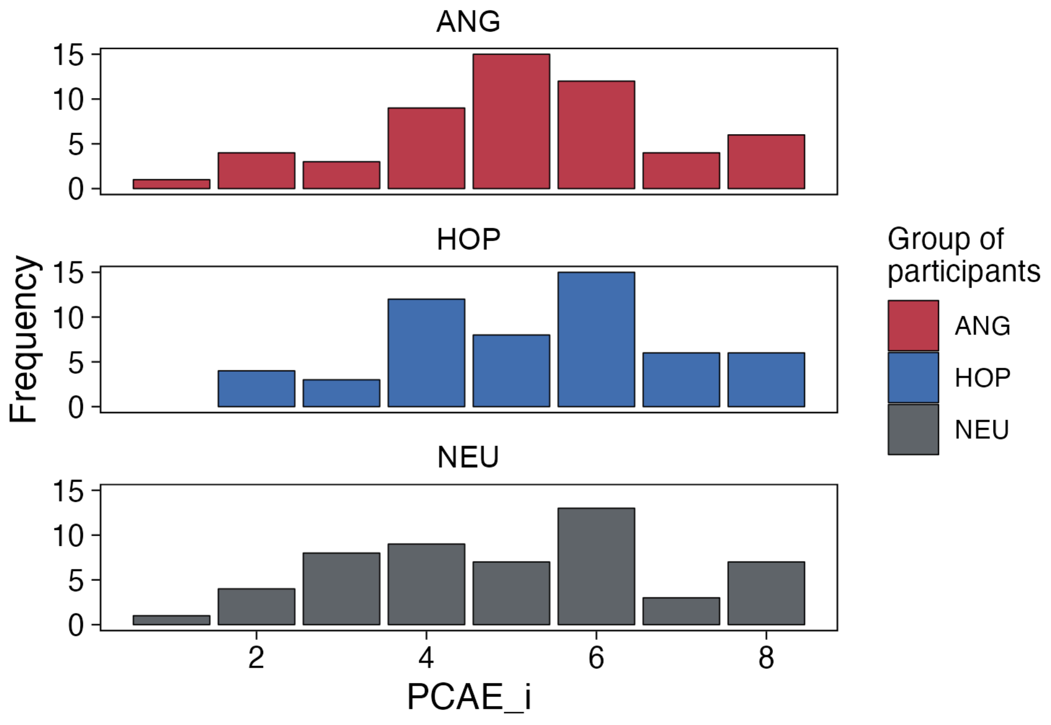

Personal and Collective Action Efficacy (Chu & Yang, 2020) - Individual efficacy - Units: Total score

Figure S13. Distribution of perceived collective climate action efficacy.

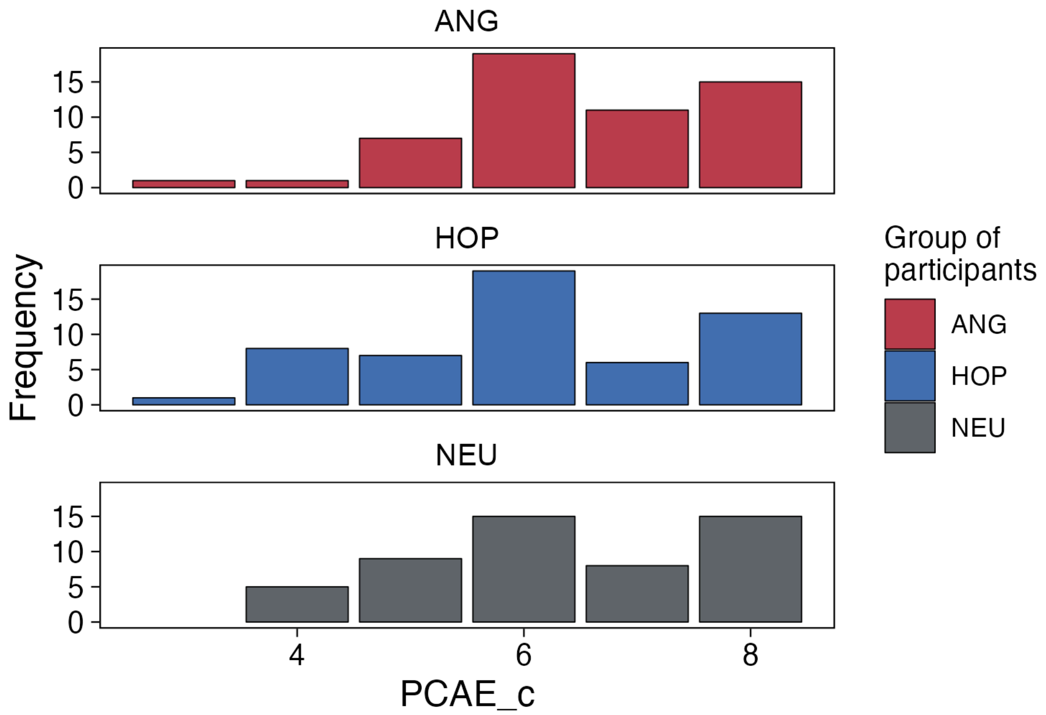

Personal and Collective Action Efficacy (Chu & Yang, 2020) - Collective efficacy
- Units: Total score

Figure S14. Distribution of psychological distance to climate change.

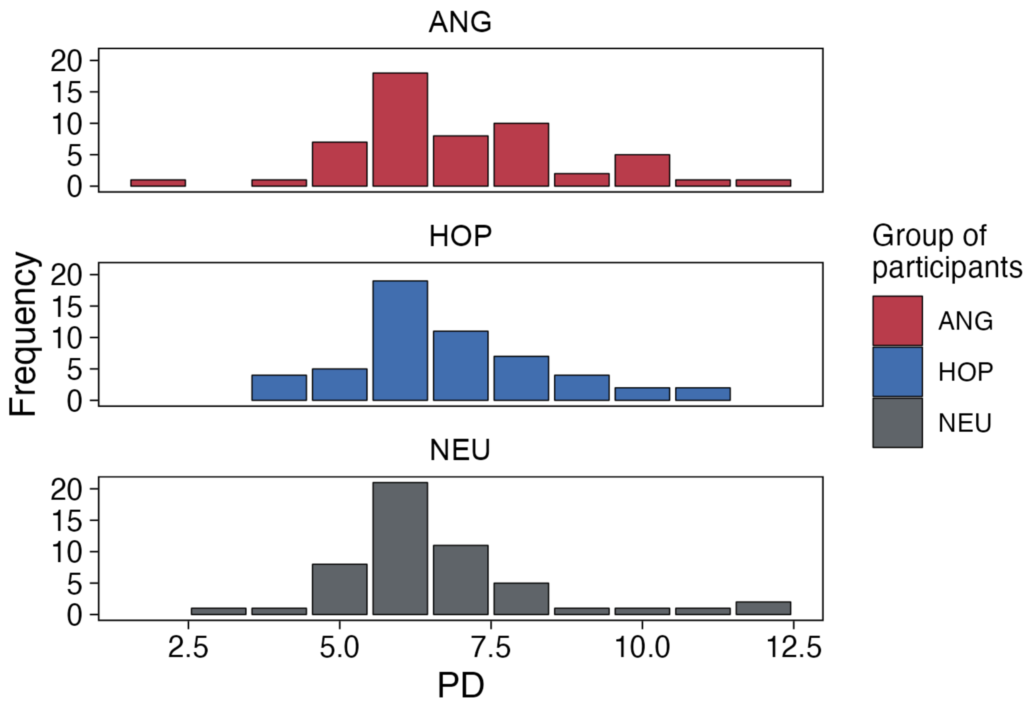

Psychological Distance to Climate Change (Valkengoed, Steg & Perlaviciute, 2021)
- Units: Total score

Figure S15. Distribution of nature relatedness across studied groups.

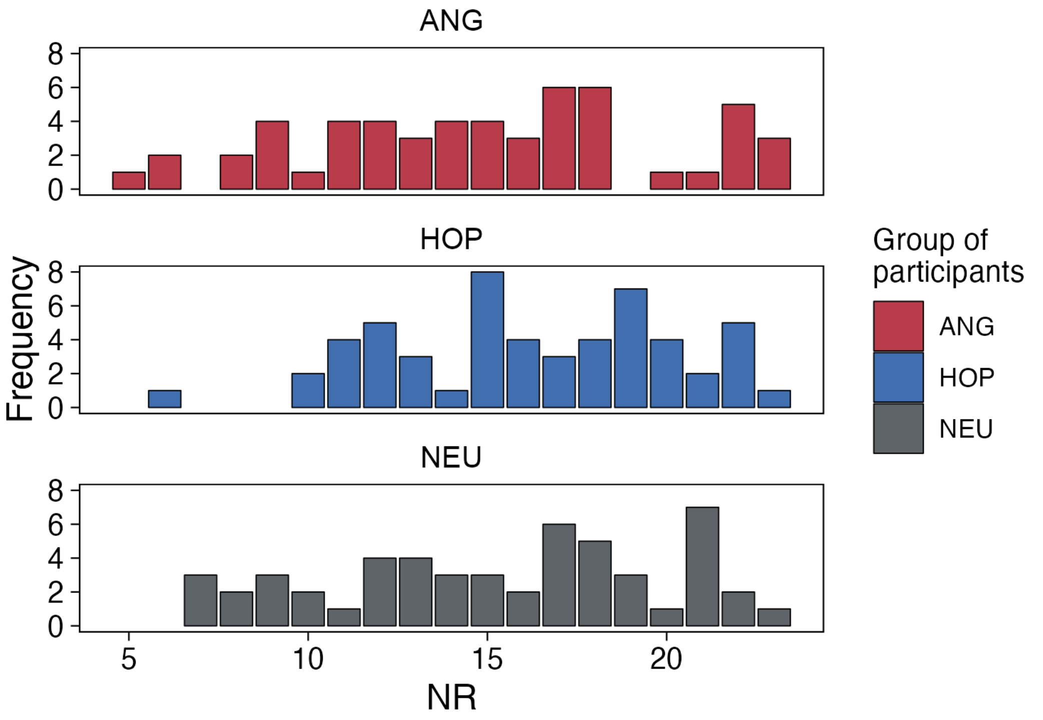

Nature Relatedness (Nisbet & Zelenski, 2013) - Units: Total score

Figure S16. Distribution of climate anger across studied groups.

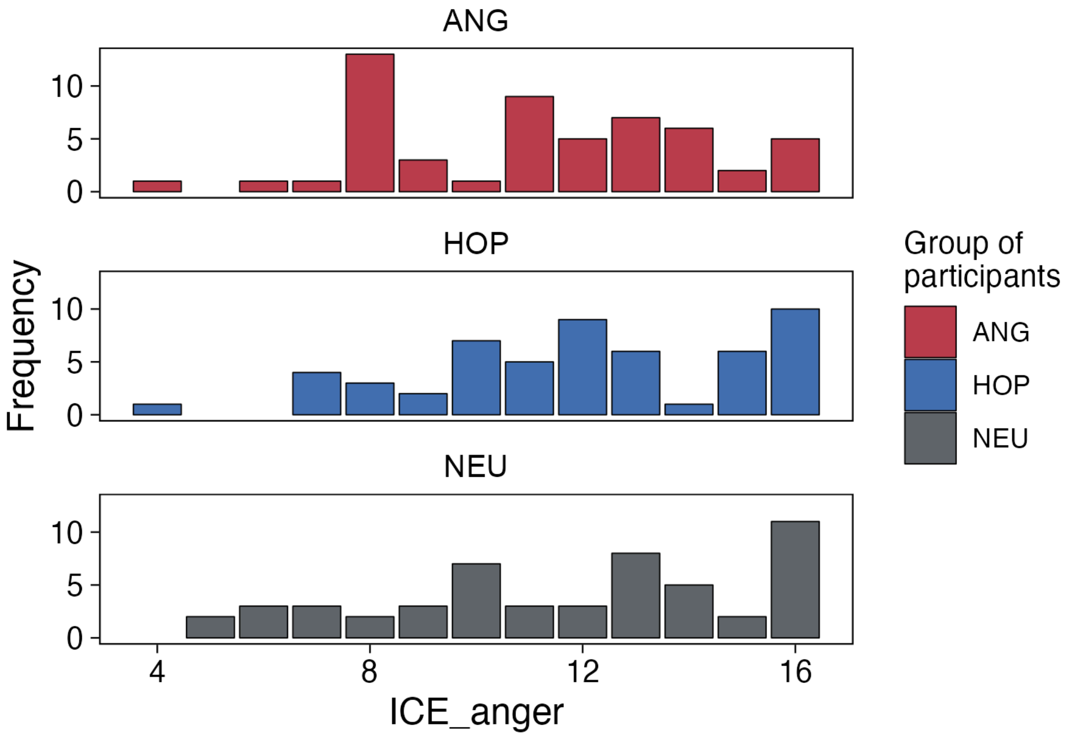

Inventory of Climate Emotions (Marczak et al. 2023) - Climate anger - Units: Total score

Figure S17. Distribution of climate anxiety across studied groups.

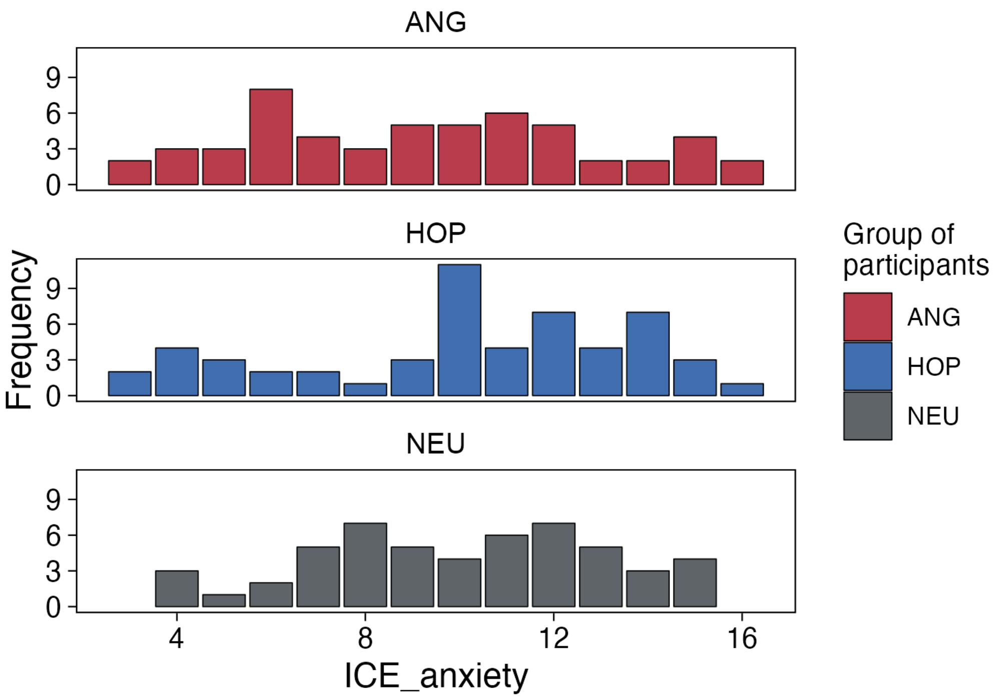

Inventory of Climate Emotions (Marczak et al. 2023) - Climate anxiety - Units: Total score

Figure S18. Distribution of climate contempt across studied groups.

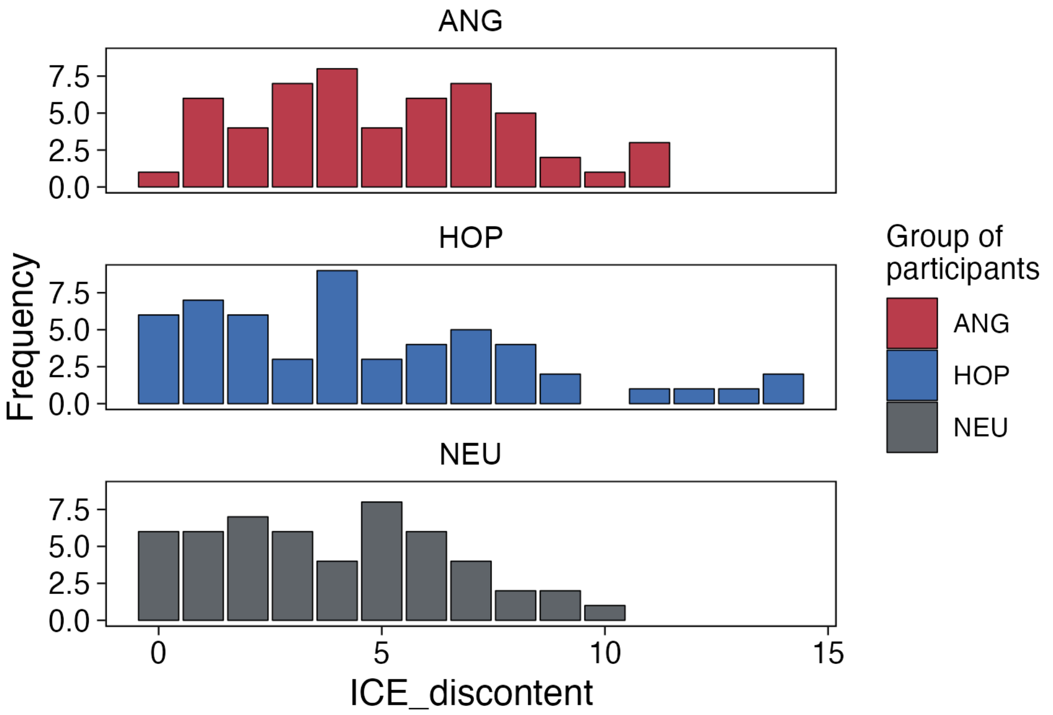

Inventory of Climate Emotions (Marczak et al. 2023) - Climate discontent - Units: Total score

Figure S19. Distribution of climate enthusiasm across studied groups.

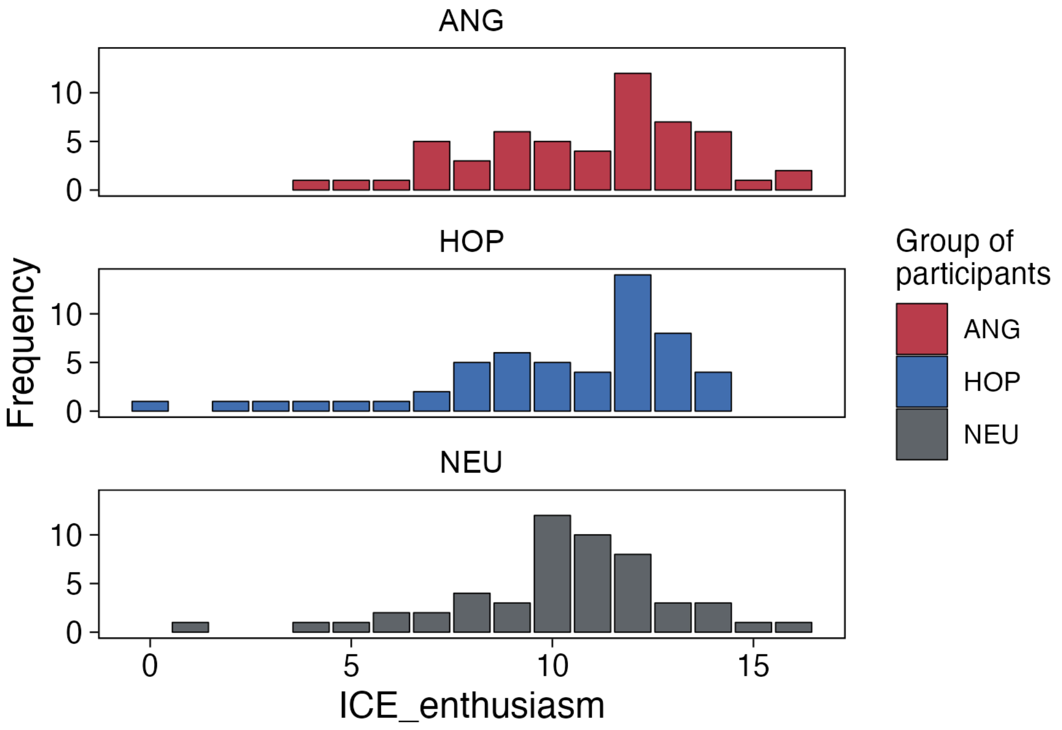

Inventory of Climate Emotions (Marczak et al. 2023) - Climate enthusiasm - Units: Total score

Figure S20. Distribution of climate guilt across studied groups.

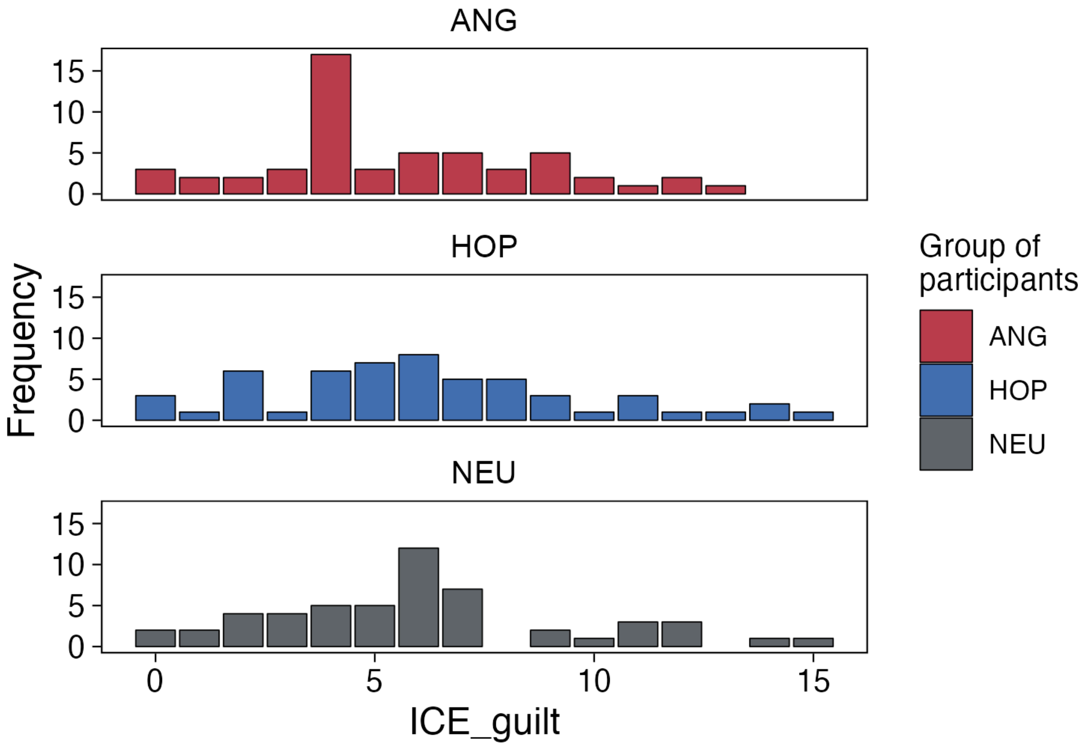

Inventory of Climate Emotions (Marczak et al. 2023) - Climate guilt - Units: Total score

Figure S21. Distribution of climate isolation across studied groups.

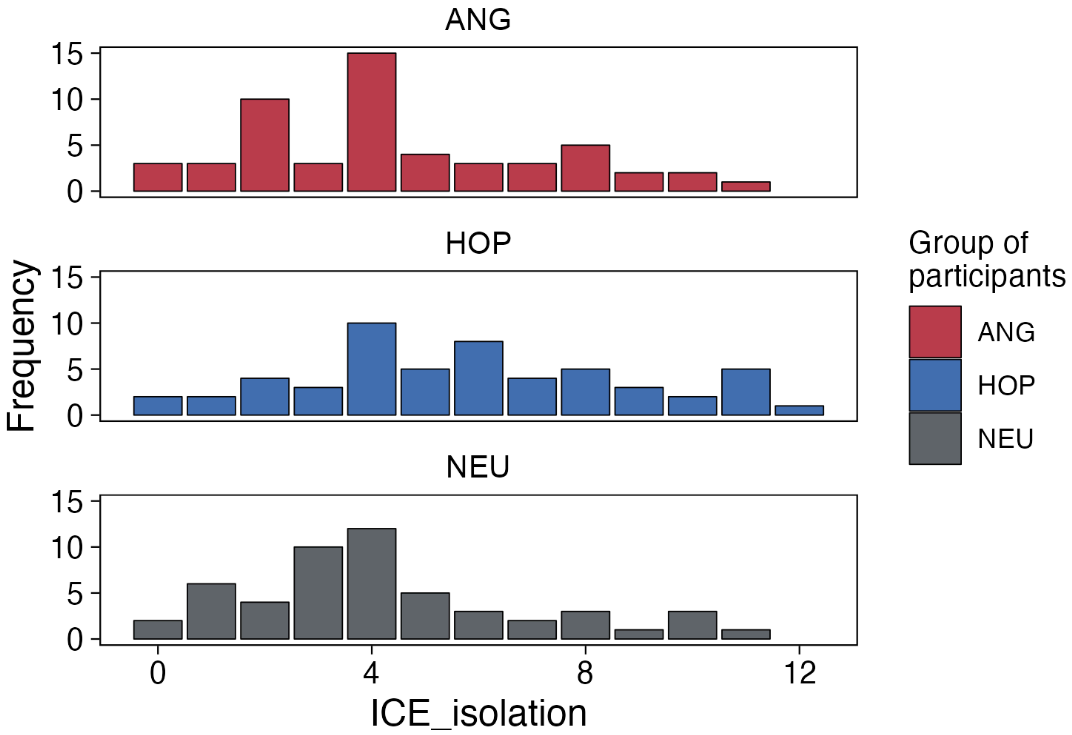

Inventory of Climate Emotions (Marczak et al. 2023) - Climate isolation - Units: Total score

Figure S22. Distribution of climate powerlessness across studied groups.

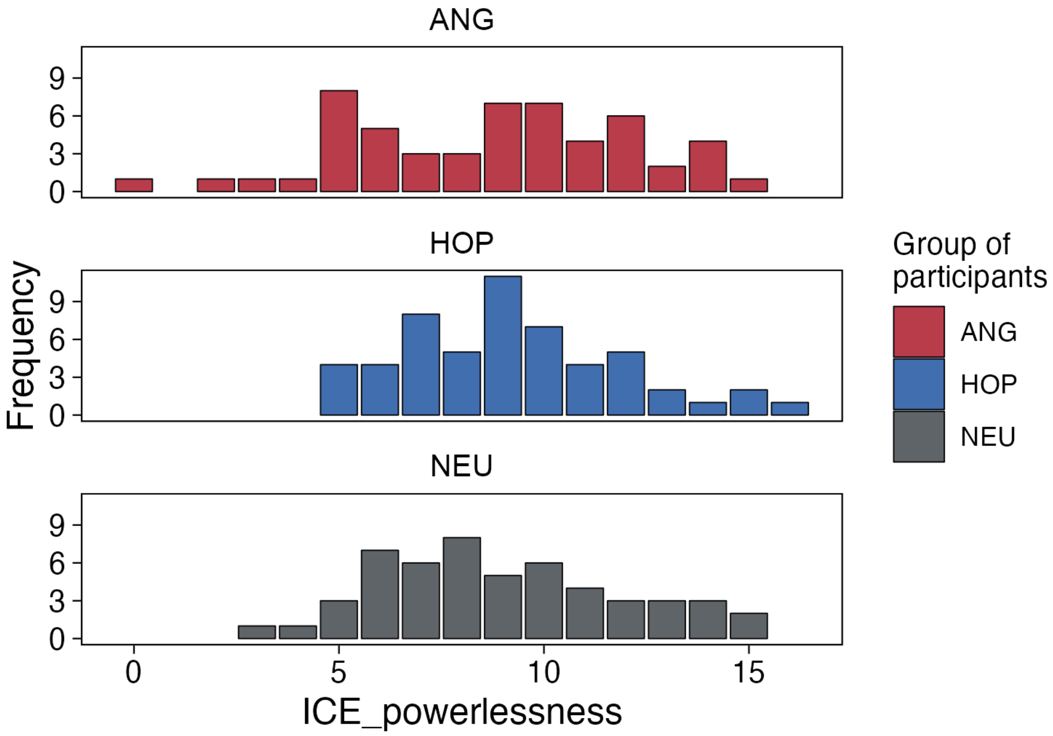

Inventory of Climate Emotions (Marczak et al. 2023) - Climate powerlessness - Units: Total score

Figure S23. Distribution of climate sorrow across studied groups.

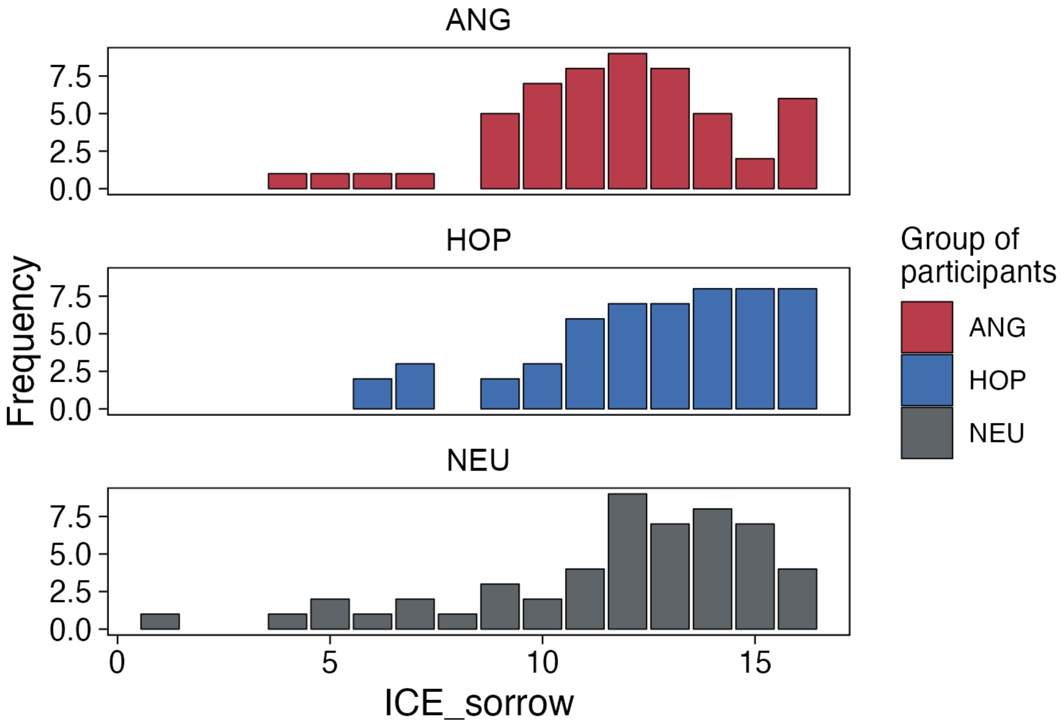

Inventory of Climate Emotions (Marczak et al. 2023) - Climate sorrow - Units: Total score

Supplementary Tables

	**fd_mean**	**fd_num**	**fd_perc**	**tsnr**
**Mean**	0.138	5.748	2.633	59.394
**Mean after exclusion**	0.126	3.889	1.738	60.194
**Min**	0.029	0	0	18.458
**Max**	1.120	212	72.143	89.589

Table S2. Summary of the quality-control (QC) metrics provided by MRIQC. Mean QC metrics are provided for the whole sample (n = 160) and after exclusion (n = 154). *Note*: fd - framewise displacement, tsnr - temporal signal-to-noise ratio.
